# MRI to guide clinical management of rectal cancer: updated consensus recommendations from the European Society of Gastrointestinal and Abdominal Radiology (ESGAR)—PART I primary staging

**DOI:** 10.1007/s00330-025-12274-w

**Published:** 2026-01-29

**Authors:** ESGAR Rectal Imaging Guideline Group, Juan-Ramón Ayuso, Svetlana Balyaniskova, Regina G. H. Beets-Tan, Ivana Blazic, Lennart Blomqvist, Damiano Caruso, Filippo Crimì, Luís Curvo-Semedo, Raphaëla C. Dresen, Marc J. Gollub, Vicky Goh, Kirsten Gormly, Sofia Gourtsoyianni, Bengi Gurses, Christine Hoeffel, Andreas M. Hötker, Natally Horvat, Davide Ippolito, Seung Ho Kim, Andrea Laghi, Max J. Lahaye, Doenja M. J. Lambregts, Monique Maas, Stephanie Nougaret, Cinthia D. Ortega, Emilio Quaia, Søren R. Rafaelsen, Pablo Rodríguez Carnero, Inês Santiago, Saugata Sen, Soleen Stocker-Ghafoor, Jaap Stoker

**Affiliations:** 1 Hospital Clinic, Barcelona, Spain; 2 Kingston and Richmond NHS Foundation Trust, London, UK; 3 The Netherlands Cancer Institute, Amsterdam, The Netherlands and GROW Research Institute for Oncology & Reproduction, University of Maastricht, Maastricht, The Netherlands; 4 Clinical Hospital Centre Zemun, Belgrade, Serbia; 5 Karolinska Institutet, Stockholm, Sweden; 6 Department of Medical Surgical Sciences and Translational Medicine, Sapienza University of Rome, Rome, Italy; 7 University of Padova, Padova, Italy; 8 Department of Imaging, Local Health Unit-Aveiro Region and Faculty of Medicine, University of Coimbra, Coimbra, Portugal; 9 University Hospitals Leuven, Leuven, Belgium; 10 Memorial Sloan Kettering Cancer Center, New York, NY USA; 11 School of Biomedical Engineering & Imaging Sciences, King’s College London, London, UK; 12 The University of Adelaide, Adelaide, SA Australia; 13 1st Department of Radiology NKUA, Areteion Hospital, Athens, Greece; 14 Department of Radiology, Koc University School of Medicine, Istanbul, Turkey; 15 Université de Reims, Champagne-Ardennes, CHU Reims, CRESTIC, Reims, France; 16 University Hospital Zürich, Zürich, Switzerland; 17 Mayo Clinic, Rochester, NY USA; 18 School of Medicine and Surgery, University of Milan-Bicocca, Monza, Italy; 19 Inje University Haeundae Paik Hospital, Busan, Korea; 20 Humanitas University, Department of Biomedical Sciences, and IRCCS Humanitas Research Hospital, Radiology Department, Milan, Italy; 21 Montpellier Cancer Center, PINKCC Lab, Montpellier, France; 22 Hospital das Clinicas HCFMUSP, Faculdade de Medicina, Universidade de Sao Paulo and Hospital Israelita Albert Einstein, Sao Paulo, Brazil; 23 University Hospital of Southern Denmark, Vejle, Denmark; 24 La Princesa University Hospital, Madrid, Spain; 25 Hospital da Luz, Lisbon, Portugal; 26 Tata Medical Center, Kolkata, India; 27 Amsterdam University Medical Centre, Amsterdam, The Netherlands

**Keywords:** Rectal cancer, Magnetic resonance imaging, Clinical guidelines, Primary staging

## Abstract

**Objectives:**

To provide up-to-date consensus recommendations on the acquisition, interpretation and reporting of MRI for the primary staging of rectal cancer.

**Materials and methods:**

A panel of twenty-six abdominal imaging experts from the European Society of Gastrointestinal and Abdominal Radiology (ESGAR) engaged in an online consensus process, led by three non-voting chairs. The process adhered to an adapted version of the RAND-UCLA appropriateness method. A total of 126 items were scored (22 general, 55 on primary staging, 49 on restaging after neoadjuvant treatment) and classified using ≥ 80% as the cut-off to establish consensus.

**Results:**

Consensus was reached for 121 items (96%). The current manuscript addresses the resulting general recommendations and those focused on baseline staging. Key updates compared to the previous guideline editions include more detailed recommendations for image acquisition, adoption of the sigmoid take-off as a landmark to discern rectal from sigmoid cancer, updated definition of mesorectal fascia involvement by a distance of ≤ 1 mm, including involvement by irregular nodes and extramural vascular invasion; a transition to a patient-level approach for cN-category assessment with updated criteria for lateral nodes including a ≥ 7 mm size threshold, and recommendations on the limited use of DWI for primary staging.

**Conclusions:**

These updated expert consensus recommendations serve as clinical guidelines for the primary staging of rectal cancer using MRI. Recommendations for restaging and response evaluation after neoadjuvant treatment are addressed in a separate publication.

**Key Points:**

***Question*** *Since the last ESGAR rectal imaging guideline update, the rectal cancer treatment landscape has further evolved, necessitating updates to the existing guidelines*.

***Findings*** *An online consensus process involving 26 panellists led to 96% consensus across 121 items discussed, including 22 general items and 55 related to primary staging*.

***Clinical relevance*** *Key updates related to primary staging include more detailed recommendations for image acquisition, adoption of the sigmoid take-off, refined criteria for MRF involvement, a new patient-level approach for cN-assessment, and recommendations on the limited use of DWI*.

**Graphical Abstract:**

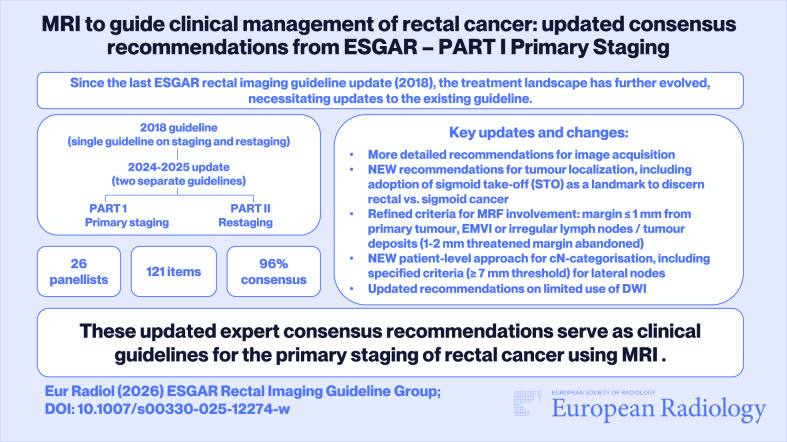

## Introduction

In 2013, the European Society of Gastrointestinal and Abdominal Radiology (ESGAR) published its first consensus recommendations on MRI for the clinical management of rectal cancer [1]. A first update was organised in 2016 (results published in 2018), introducing structured template reports for primary (baseline) staging and restaging after neoadjuvant treatment, as well as practice recommendations on lymph node staging and the use of diffusion-weighted imaging (DWI) [2].

Since these last updates, the treatment landscape of rectal cancer has further evolved. Major developments include the growing adoption of organ-preserving treatment strategies and the introduction of new neoadjuvant treatment regimens, including total neoadjuvant treatment. At the same time, there has been a gradual shift away from traditional tumour nodes metastases (TNM) based staging toward more tailored methods of risk stratification, focusing on the presence of high-risk factors such as extramural venous (or vascular) invasion (EMVI). Other recent developments include the introduction of the sigmoid take-off (STO) as an anatomical landmark to define the upper boundary of the rectum, and the increased use of artificial intelligence (AI) and quantitative image analysis tools such as Radiomics.

This guideline update aims to incorporate these innovations and provide a state-of-the-art framework for the acquisition, interpretation, and reporting of rectal MRI. The current paper focuses on MRI for the primary staging of newly diagnosed rectal cancer. Recommendations for response evaluation and restaging after neoadjuvant treatment (resulting from the same guideline process) are addressed in a separate publication [3].

## Materials and methods

A modified version of the RAND-UCLA appropriateness method was chosen as the methodology for this update. The guideline development process, which follows the recommendations outlined by the ESGAR research committee [4], is summarised as follows:

### Step 1—Panel selection

The ESGAR research committee appointed three radiologists (D.L., M.M., R.B.-T.) as non-voting guideline chairs. The chairs distributed a call to the full ESGAR membership to recruit additional panellists. Twenty-six voting panellists (J-R.A., S.B., I.B., L.B., D.C., L.C-S., R.D., M.G., V.G., K.G., S.G., B.G., C.H., A.H., N.H., D.I., S.K., A.L., M.L., S.N., C.O., E.Q., S.R., I.S., S.S., and J.S.) were selected based on their recognised expertise in rectal cancer imaging, with careful consideration given to gender balance and geographical distribution. Additionally, three junior fellows (F.C., P.R.C., and S.S.-G.) were appointed to assist with the literature review and evidence synthesis.

### Step 2—First questionnaire and voting round

The three chairs designed a first online questionnaire, using Google Forms, which was distributed to the panel on 22 March 2024. Panellists were asked to indicate whether they still agreed with each recommendation from the 2018 guideline publication or if the item should be revisited for an updated literature review and renewed panel vote. Panellists were also invited to suggest relevant topics that were not previously included.

### Step 3—Literature review and evidence synthesis

Based on the responses from Step 2, the chairs compiled a list of clinical questions to be addressed in the literature review, including topics that did not reach ≥ 80% consensus in the first voting round and additional topics suggested by the panel. The literature review was conducted by the three junior fellows, who synthesised the available evidence. The process was supervised by two of the chairs (D.L. and M.M.), who compiled a final evidence synthesis document including draft statements and corresponding levels of evidence (see Supplement 1).

### Step 4—Second questionnaire and voting round

The draft statements were formatted into an online questionnaire (using Google Forms), which was sent to the panel on 13 January 2025, together with a link to the evidence synthesis document. For each draft statement, panellists were asked to indicate whether they agreed, disagreed, or were uncertain, and to provide comments or suggestions where appropriate.

### Step 5—Data analysis and reporting

Data from the two voting rounds were compiled and analysed by D.L. and M.M., who calculated descriptive statistics. Each item was categorised as (a) appropriate/recommended (≥ 80% consensus), (b) inappropriate/not recommended (≥ 80% consensus), or (c) uncertain (lacking consensus, i.e. < 80% consensus). As only a minority of items did not reach consensus (and near-consensus was reached for all of them, detailed in the “Results” section), no additional voting round was conducted. Instead, these items were thoroughly discussed in the draft manuscript, including explanations of the panel’s comments and considerations from Step 4. The draft manuscript was distributed to the panellists on 10 May 2025 for final review and editing, after which the final version was approved by all panellists.

## Results

The voting panel included 13 male and 13 female members from 18 countries. Panellists had a median of 16 years of experience in rectal MRI, handling a median number 100 rectal MRI cases per year. Further panel details are provided in Table 1.

**Table 1:** Demographic data of the twenty-six voting panellists

		Total	%
Total		26	100%
Gender	Male	13	50%
	Female	13	50%
Country	Australia	1	4%
	Belgium	1	4%
	Brazil	1	4%
	Denmark	1	4%
	France	2	8%
	Greece	1	4%
	India	1	4%
	Italy	4	15%
	Korea	1	4%
	The Netherlands	2	8%
	Portugal	2	8%
	Serbia	1	4%
	Spain	1	4%
	Sweden	1	4%
	Switzerland	1	4%
	Turkey	1	4%
	UK	2	8%
	USA	2	8%
Main working place	Comprehensive cancer centre or dedicated oncology centre	13	50%
	Academic teaching hospital	10	38%
	Non-academic hospital/other	3	12%
Median no. of rectal cancer MRIs read per year	100 (range 45–500)		
Median years of experience in rectal MRI	16 (range 10–32)		
Working in a centre offering organ preservation (W&W)	Yes	21	81%
	No	5	19%
Institutional MDT set up	Dedicated anorectal or colorectal MDT	21	81%
	CRC cases embedded in the general oncologic MDT	5	19%
Institutional practice with respect to patient preparation	Routine use of spasmolytics	17*	71%
	Routine use of a preparatory micro-enema	9*	38%
	Routine use of rectal filling	3*	13%

* Based on available responses of 24 out of 26 panellists

*CRC* colorectal cancer, *MDT* multidisciplinary team, *W&W* Watch–and–wait

### Areas of consensus

**Fig. 1:**
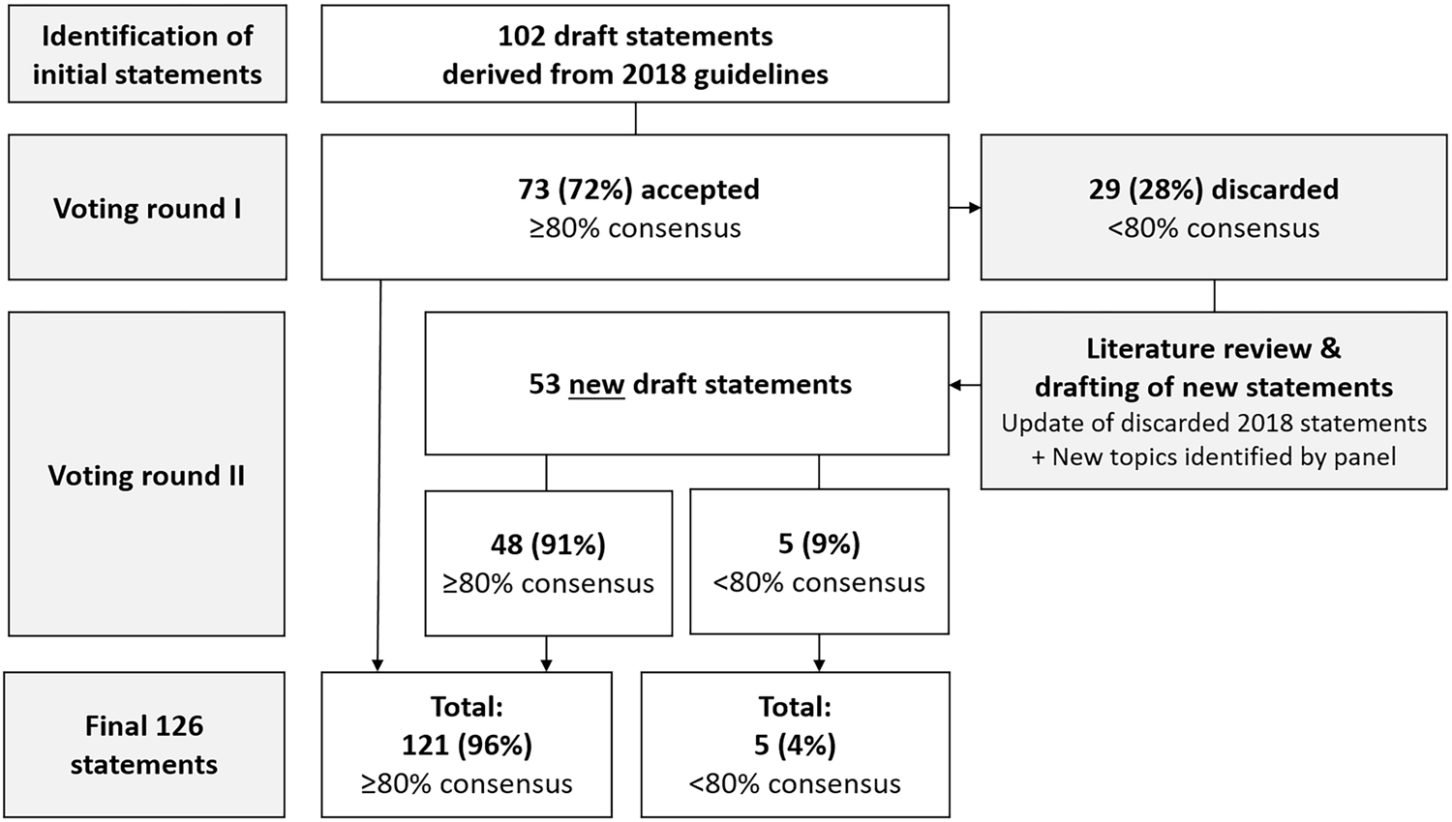
Flowchart of the guideline development process

**Fig. 2:**
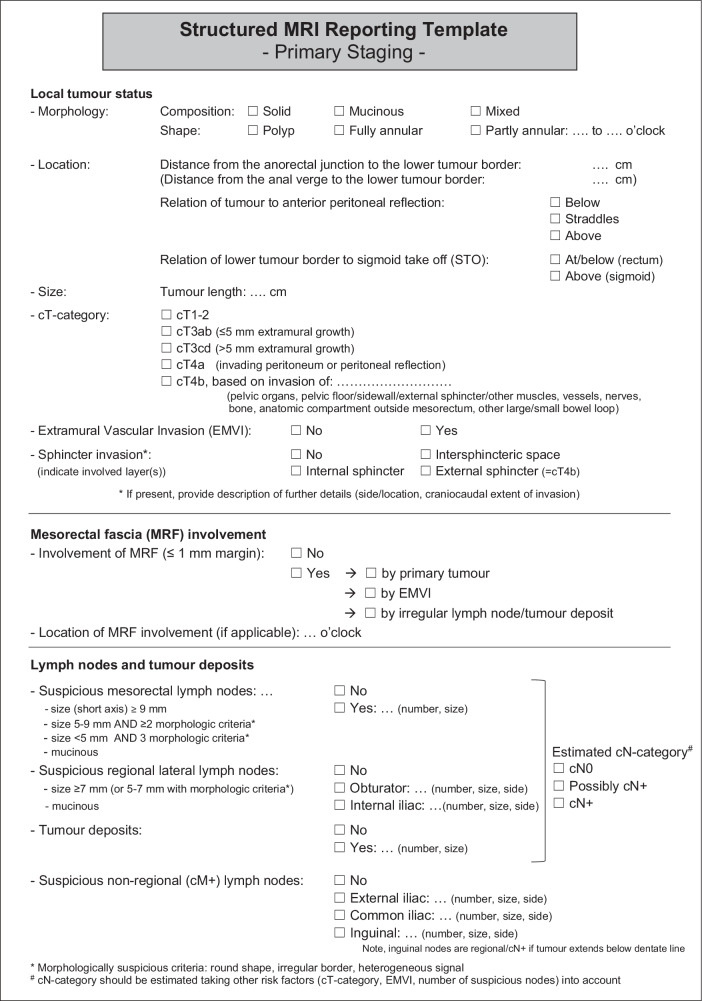
Recommended structured reporting template for primary staging

The guideline process ultimately resulted in 126 statements, for which ≥ 80% consensus was reached in 121 (96%), as outlined in Fig. 1. The following sections elaborate on the 22 general recommendations and 55 focused on primary staging. Table 2 provides a full overview of all recommendations; key updates compared to the previous guideline edition are summarised in Table 3. The recommended updated template for structured reporting is shown in Fig. 2.

**Table 2:** Synopsis and key recommendations for baseline MRI staging of rectal cancer (based on items for which ≥ 80% consensus was reached)

I—Recommendations for MR image acquisition	% Consensus
Hardware and patient preparation	
- MRI should routinely be performed for the baseline staging (before treatment) and restaging (after neoadjuvant treatment) of rectal cancer	100%
- The recommended MRI field strength is 1.5 T or 3.0 T (with no clear preference for either of the two)	92%
- MRI of rectal cancer should be performed using an external surface coil; use of an endorectal coil is not recommended	100%
- Endorectal ultrasound is the preferred technique for the differentiation and categorisation of T1 tumours	88%
- Endorectal filling is not recommended	96%
**- A preparatory micro-enema is optional**	96%
High resolution T2W	
- The standard MRI protocol for staging should include 2D T2-weighted sequences in 3 planes	100%
- Slice thickness should be 3 mm or less**; in-plane resolution should be less than 1** **×** **1** **mm**	96–100%
- 3D T2-weighted sequences are optional as an add-on, but not recommended to replace 2D T2-weighted sequences	83%
- Transverse sequences should be angled perpendicular to the rectal tumour axis; coronal sequences should be angled parallel to the rectal tumour axis	96–100%
- In distal tumours, a coronal sequence parallel to the anal canal should be included to properly assess the relation between the tumour and anal sphincter	100%
DWI	
- The standard MRI protocol for primary staging should include a DWI sequence	88%
- A DWI sequence should include at least one high b-value of b800 or higher	96%
- DWI sequences, including their corresponding ADC maps, should be primarily assessed visually (quantitative ADC measurements are not recommended for clinical staging)	92%
**- DWI should be acquired in the same plane as the axial T2-weighted sequence (oblique-axial, perpendicular to the tumour axis)**	92%
**- Reduced FOV DWI is recommended (in particular for restaging); full FOV acquisitions are optional to ensure complete assessment of the whole pelvis, including all pelvic LN stations**	81%
Other	
**- The protocol should include at least one large FOV T2W (or alternatively T1W) sequence covering all pelvic compartments from the level of the aortic bifurcation/promontory to the distal margin of the anal canal including the inguinal lymph node regions; this sequence may either be acquired as part of the high resolution protocol or added as a fast-acquisition sequence (e.g. single shot fast spin echo or turbo spin echo)**	92%
- Non-enhanced T1W sequences are not mandatory for staging	88%
- Non-dynamic contrast-enhanced sequences and fat-suppressed sequences are not mandatory for staging	83%
- Dynamic contrast-enhanced sequences are not mandatory for staging	96%
- Fat-suppressed sequences are not mandatory for staging	83%

II—Recommendations for MR image interpretation and reporting	
General	
- Structured reporting of rectal cancer MRI is recommended (and should include the items described in the structured reporting template in Fig. 2).	96%
**- Radiomics and/or deep learning models should not be used to guide clinical decision making**	96%
Tumour location	
**- It is recommended to include the relation of the tumour to the STO in the MRI report to discern rectal from sigmoid cancers**	81%
**- It is recommended to classify tumours with a lower tumour border starting below the STO as rectal, and tumours starting above the STO as sigmoid**	92%
Tumour (cT-) category	
- A tumour that invades the MRF (but no other organs or structures) should be categorised as cT3 MRF+ and not cT4	92%
**- cT-category should primarily be informed by the extent of tumour invasion at the level of the rectum; involvement of the internal anal sphincter and intersphincteric space should not be taken into account in cT-categorisation**	85%
**- Involvement of the external anal sphincter should be categorised as cT4b**	96%
- A tumour that invades the pelvic floor muscles or pelvic sidewall muscles should be categorised as cT4b	100%
Mesorectal fascia (MRF) and peritoneum	
**-** **A margin of** ≤ **1** **mm between the MRF and the primary tumour, EMVI, or irregular nodes (representing tumour deposits or lymph nodes with extracapsular extension) is recommended as a criterion to diagnose an involved MRF**	100%
**-** **The MRF should be considered as non-involved in case of a** ≤ **1** **mm margin from lymph nodes (non-enlarged or enlarged) with smooth margins**	96%
**- Reporting of a ‘threatened’ MRF (distance 1–2** **mm) is not recommended**	92%
- Anterior invasion above the level of the peritoneal reflection entails peritoneal (and not MRF) invasion	100%
Lymph nodes and tumour deposits (cN-category)	
**-** **Nodal category should be reported as cN0 or cN+ and include a level of confidence (definitely cN0; possibly cN**+**; definitely cN**+**)**	92%
**- Known risk factors associated with N+ disease (e.g. higher cT-category, presence of EMVI, higher number of nodes) should be taken into account when determining the risk for N+ disease**.	89%
- The nodal staging criteria proposed by ESGAR in 2018 (which combine size and morphology) are still recommended to evaluate individual nodes	85%
**-** **A size threshold of** ≥ **7** **mm (short-axis diameter) is recommended to diagnose malignant nodes in the obturator and internal iliac compartments**	89%
**- The presence of malignant morphologic features (round shape, internal heterogeneity, loss of fatty hilum, and border irregularity) supports the suspicion of malignancy in intermediate-sized (5–7** **mm) lateral lymph nodes**	92%
**- Elongated nodes immediately dorsal to the external iliac veins should be considered benign**	100%
**- Mesorectal, obturator and internal iliac nodes are part of the N-category; M-category lymph nodes include the external iliac and common iliac nodes; Inguinal lymph nodes are part of the M-category, except in tumours involving the anal canal below the dentate line, in which case they are part of the N-category**	96%
**- All suspicious nodes (whether regarded as lymph nodes or tumour deposits) should be combined to determine the cN-category on MRI**	81%
**- Recommended criteria to diagnose mesorectal nodes as tumour deposits and discern them from lymph nodes include irregular shape and contiguity with veins**	92%
Extramural vascular invasion (EMVI)	
**- Grade 3 and grade 4 EMVI should be regarded as EMVI positive**	100%
Use of DWI	
**- DWI should be assessed in conjunction with T2W MRI**	100%
- DWI is not recommended for baseline cT-categorisation, assessment of EMVI or MRF involvement	89%
**- Use of DWI is optional to detect and localise lymph nodes, but DWI is not recommended for characterisation of lymph nodes or to differentiate between lymph nodes and tumour deposits**	100%

Note, recommendations in **bold** font represent recommendations that have been updated or newly added compared to the previous guidelines edition

*ADC* apparent diffusion coefficient, *DWI* diffusion–weighted imaging, *EMVI* Extramural venous invasion/extramural vascular invasion, *FOV* field of view, *LN* lymph node, *MRF* Mesorectal fascia, *STO* sigmoid take off

**Table 3:** Overview of main changes and additions to the previous guideline editions

Topic	Updated recommendation*	Old recommendation (from 2016 consensus meeting)
Patient preparation	• A preparatory micro-enema is optional	• Use of an enema is not routinely recommended
	• Spasmolytics are particularly recommended for mid-high rectal tumours or in patients with small bowel loops descending low in the pelvis in whom bowel movement artefacts are more likely to occur (77% consensus)	• Spasmolytics may be useful to reduce bowel movement artefacts (57% consensus)
MR protocol and acquisition	• In-plane resolution for T2W MRI should be < 1 × 1 mm	• N/A (New recommendation)
	• DWI should be angled in the same oblique-axial plane as T2W MRI	• N/A (New recommendation)
	• Reduced FOV DWI is recommended (in particular for restaging); full FOV acquisitions are optimal for whole-pelvic imaging	• N/A (New recommendation)
	• The protocol should include a large FOV T2W or T1W sequence covering all relevant pelvic lymph node compartments	• N/A (New recommendation)
Tumour localisation	• The relation of the tumour to the sigmoid take off (STO) should be reported; tumours with a lower border starting below the STO should be classified as rectal, and tumours starting above the STO as sigmoid	• N/A (New recommendation)
	• Uniform definitions for tumour height measurements and location classification are recommended, applied consistently within institutions. Although landmark choice may vary—with some preferring the anal verge—ESGAR suggests, with 77% consensus, a two-category system classifying tumours as distal (starting ≤ 5 cm from anorectal junction) or mid-high (starting > 5 cm from anorectal junction), using one or more straight measurements along the centre of the rectal lumen	• N/A (New recommendation)
cT-category in distal tumours	• cT-category should primarily be defined at the level of the rectum; involvement of the internal sphincter and the intersphincteric space does not impact cT-categorisation	• N/A (New recommendation)
	• Involvement of the external anal sphincter is classified as cT4b	• No consensus reached in previous guidelines (71%)
MRF	• MRF+ is defined as a ≤ 1 mm margin between the MRF and the primary tumour, EMVI, or irregular lymph nodes/TDs; a ≤ 1 mm margin from lymph nodes (regardless of size) with smooth margins should be considered MRF-	• Previous recommendations did not detail how to handle MRF involvement by EMVI, lymph nodes and TDs
	• Reporting of a threatened MRF (1–2 mm margin) is no longer recommended	• The previous reporting template included a threatened (1–2 mm) subcategory
Lymph nodes and tumour deposits (cN-category)	• Recommended patient-level approach for cN-categorisation, taking into account size and morphologic criteria for individual nodes and combining these with other risk factors for N+ disease (higher cT-category, EMVI, higher number of nodes) to classify the cN-category as cN0, possibly cN+ or cN+	• Patient-level approach, including a confidence level, is a new recommendation. Criteria to assess individual mesorectal lymph nodes were adopted from previous guidelines
	• Lateral lymph nodes: - Recommended size threshold of ≥ 7 mm to define cN+ for obturator and internal iliac lymph nodes - Acknowledgement of morphologic features to support the diagnosis of cN+ in 5–7 mm nodes - Recommendation to consider elongated nodes immediately dorsal to external iliac veins as cN0	• N/A (New recommendation)
	• Recommended definitions for regional/cN-category lymph nodes (mesorectal, obturator, internal iliac, and inguinal in tumours below the dentate line) and non-regional/M-category pelvic lymph nodes (external and common iliac, inguinal—unless tumour below dentate line)	• N/A (new recommendation)
	• All suspicious nodules (whether lymph nodes or TDs) should be combined to define the cN-category; recommended criteria to discern TDs from lymph nodes include irregular shape and contiguity with veins	• N/A (new recommendation)
EMVI	• Grade 3 and Grade 4 EMVI should be considered as EMVI+	• N/A (New recommendation)
DWI	• DWI should be assessed in conjunction with T2W MRI	• N/A (New recommendation)
	• DWI is optional to detect lymph nodes, but is not recommended for nodal characterisation or to discern between lymph nodes and TDs	• New recommendation. The statement that DWI is not recommended for cT-categorisation, EMVI or MRF assessment was adopted from previous guidelines

* Unless otherwise indicated, recommendations presented in this Table achieved ≥ 80% consensus

*DWI* diffusion–weighted imaging, *EMVI* Extramural venous invasion/extramural vascular invasion, *FOV* field of view, *MRF* Mesorectal fascia, *STO* sigmoid take off, *TD* Tumour deposit

### Areas lacking consensus

For 5/126 items (4%) < 80% consensus was reached. Notably, near-consensus (77% agreement) was attained for each of these topics. Reasons for non-consensus and further panel considerations are highlighted in Table 4. Topics included the use of spasmolytics, reporting of tumour deposits (TDs), and methodology for measuring tumour height and classifying tumour location.

**Table 4:** Overview of near-consensus areas

	Original statements*	Rationale, comments and recommendations from the panel
Use of spasmolytics	- Spasmolytics are optional, especially for upper rectal tumours or in patients with small bowel loops descending low in the pelvis, in whom bowel movement artefacts are more likely to occur	- Some panellists (15%) consider spasmolytics mandatory, especially considering the high safety profile with minimal side effects. The majority of panellists (71%) apply them routinely. - The panel agreed that spasmolytics are particularly recommended for mid-high rectal tumours or in patients with small bowel loops descending low in the pelvis, in whom bowel movement artefacts are more likely to occur
Reporting of TDs	- N1c category (presence of only TDs in the absence of any malignant nodes) cannot be reliably established on MRI - A prose description of the suspected presence of TDs should be included in the report and conclusion	- TDs have worse prognostic implications than cN+ lymph nodes, but no conclusive evidence is available on whether MRI can reliably distinguish between the two. - Some panellists questioned the clinical impact of reporting TDs separately, considering these inaccuracies, while others felt that, in some clear-cut cases, it may be feasible to discern them and suggest N1c status. The panel agreed that—for now—all suspicious nodules (whether regarded as lymph nodes or TDs) should be combined to determine the cN-category as cN0/cN+ as outlined in the structured reporting template and in Fig. 3. Most panellists agreed that a separate description detailing the (potential) presence of TDs should be included in the report, in addition to the cN-category
Tumour localisation and height measurements	- There are no evidence-based definitions on how to measure tumour height or classify tumours as distal, mid or high. - Aiming to enhance uniformity, ESGAR recommends the following: 1. Measure the distance between the lower tumour border and the anorectal junction (the distance from the anal verge may be reported as an add on) 2. Distal = tumours with a lower border starting < 5 cm from the anorectal junction 3. Mid-high = tumours with a lower border starting ≥ 5 cm from the anorectal junction 4. Measurements should be performed using one or more straight lines following the centre of the lumen	Main reasons for non-consensus concerned the choice of landmarks and cut-offs:- - Some panellists (8%) preferred the anal verge and some (8%) suggested alternative cut-offs (4 cm from anorectal junction or 5 cm from anal verge) - Some panellists (8%) preferred to use anatomic landmarks (rather than measurements) to discern distal, mid and high tumours - The panel agreed that uniform definitions are beneficial to improve consistency in MDT discussions and treatment guidance. While landmark (and cut-off) preference may vary per institution, it should be clearly defined and applied consistently to avoid variations, such as in- or excluding the 3–5 cm long anal canal in the height measurements

* The statements presented in this Table each reached 77% consensus

*MDT* multidisciplinary team, *TD* Tumour deposit

### Main changes and additions

**Fig. 3:**
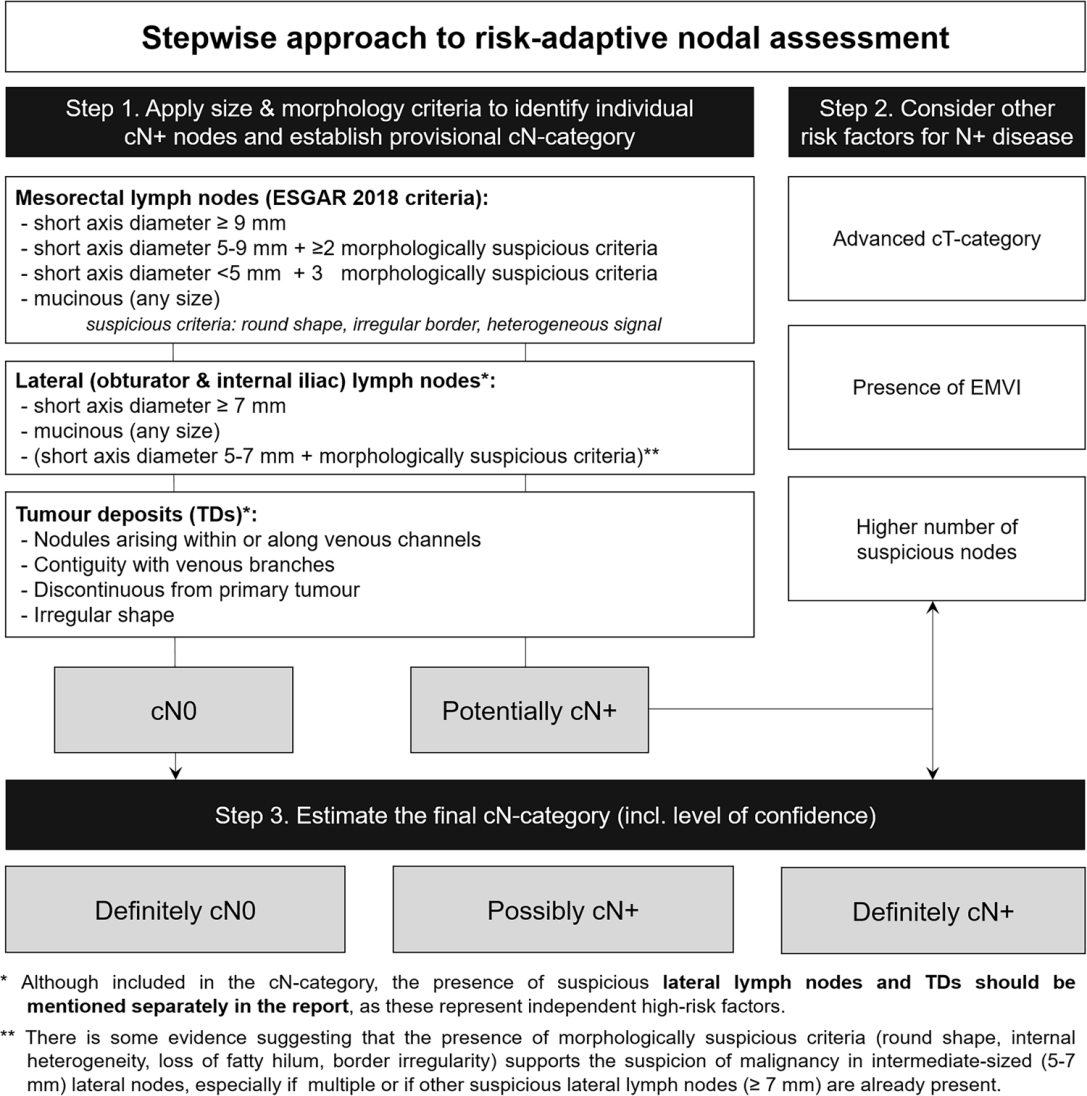
Stepwise flowchart to estimate the overall risk for N-positive disease, while acknowledging the uncertainties associated with MRI for nodal assessment. EMVI, Extramural venous invasion/extramural vascular invasion

**Fig. 4:**
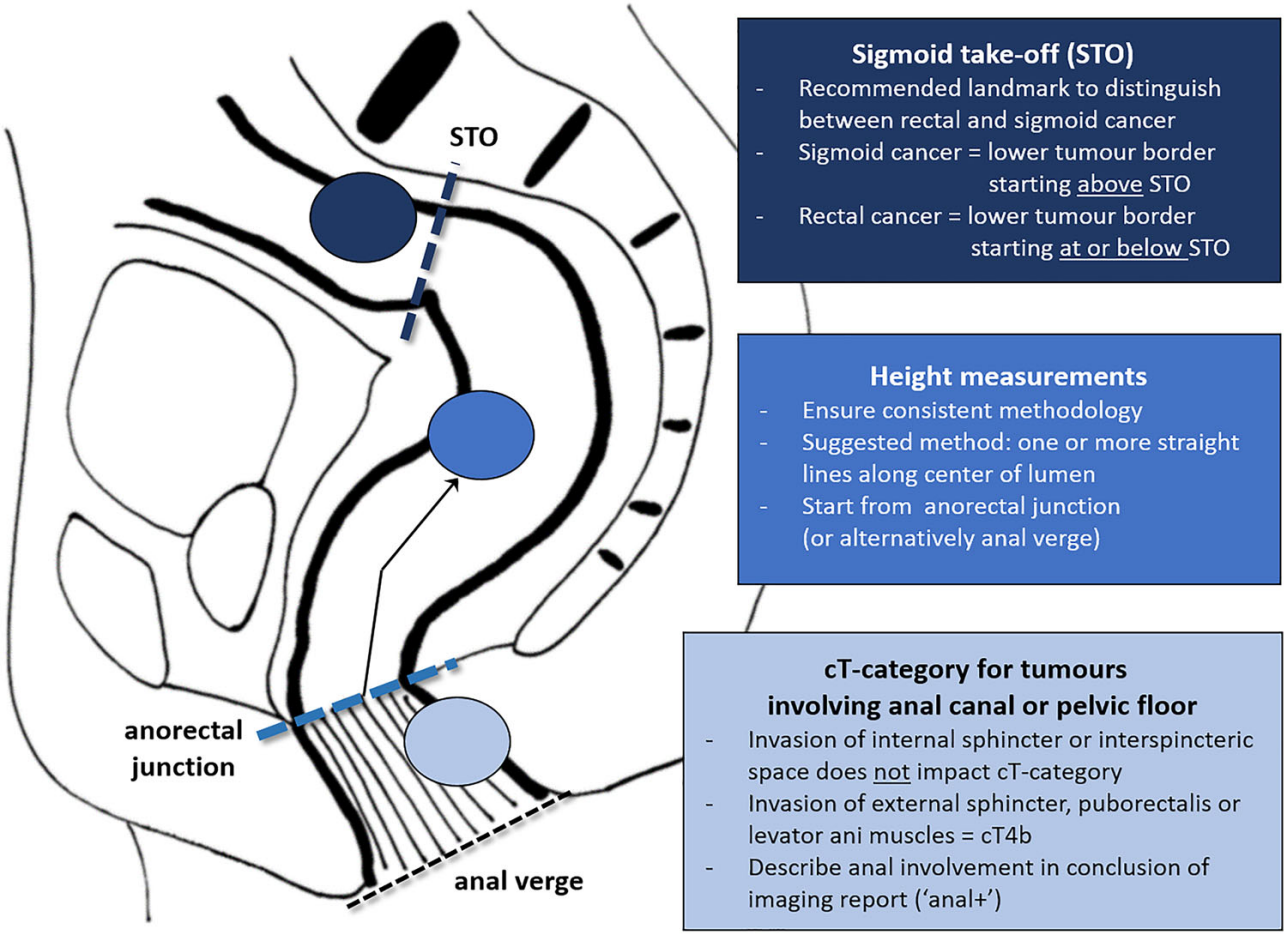
Overview of key recommendations related to tumour localisation and categorisation

Key updates compared to the previous consensus meeting results published in 2018 (see Table 3) included more detailed recommendations for image acquisition, updated criteria to localise tumours, refined criteria to assess MRF involvement, and a revised patient-centred approach for cN-categorisation (illustrated in detail in Fig. 3). A summary of recommendations regarding tumour localisation, including the adoption of the STO, guidance on tumour height measurements, and cT-categorisation in distal tumours, is provided in Fig. 4.

## Discussion

### Imaging techniques

Consistent with previous editions, the panel confirmed that MRI remains the modality of choice for local staging of rectal cancer. Although some recent data suggest that high-resolution MRI may identify early tumours eligible for local excision [5], the majority of the panel agreed that endorectal ultrasound remains preferred to stage early tumours, given its superior performance compared to MRI [6]. Additionally, advanced endoscopic techniques, such as magnifying chromoendoscopy, can be useful for evaluating tumour depth in early (T1) colorectal cancers [7].

High-resolution T2W sequences with slice thickness ≤ 3 mm remain mandatory for staging. The updated guidelines now define high resolution as an in-plane resolution < 1 × 1 mm. Although not further specified by the panel, as a ‘rule of thumb’, a resolution of 0.6 × 0.6 mm may be considered, in line with definitions from the MERCURY group and some recent international guidelines [8–10].

A new recommendation emphasises including at least one large field of view (FOV) T2W or T1W sequence covering the entire pelvis—from the aortic bifurcation to the distal anal canal, including the inguinal nodes—to ensure complete assessment of relevant compartments.

### Patient preparation

Contrary to the 2018 guidelines, the panel reached consensus that endorectal filling is not recommended, as existing literature shows no improvement in staging accuracy [11, 12]. Rectal distension may compress surrounding structures, potentially altering the distance between the rectum and critical landmarks such as the MRF, although no conclusive data links this to reduced accuracy. Near-consensus was reached on the use of spasmolytics: 77% of the panel supported their use, particularly for upper rectal tumours or in patients with low-lying small bowel loops, where bowel movement artefacts are more likely. Spasmolytics are routinely administered by 71% of the panel in their clinical practice.

For the first time, recommendations on preparatory micro-enemas were included. The panel agreed that a micro-enema is optional for baseline staging but recommended for restaging to minimise endoluminal gas and reduce susceptibility artefacts on DWI [13, 14].

### Definitions for rectal tumour localisation

Previous guideline editions did not define the upper and lower boundaries of the rectum. In 2019, an international multidisciplinary expert panel agreed on the STO as the preferred landmark to distinguish the rectum from the sigmoid colon [15]. On MRI, the STO appears as the point where the sigmoid sweeps horizontally (away from the sacrum) on sagittal views and ventrally on axial views. Evidence suggests the STO is a moderately to well-reproducible landmark to discern rectal cancers (lower tumour border starting below or at the STO) from sigmoid cancers (above the STO), though some interpretation pitfalls have been described [16, 17]. Evidence, primarily from the Netherlands and China, indicates that using the STO may impact treatment planning in 20-30% of cases, compared to traditional landmarks [16, 18–20].

The 2018 ESGAR structured reporting template included the anorectal junction as the key landmark to measure rectal tumour height [2]. However, as highlighted by Goedegebuure et al [21], there is significant variation across international guidelines in both landmarks and measurement cutoffs. Most adopt a 3-way classification (distal, mid, high), while some use a two-way classification (distal or high). Given that mainly distal location impacts treatment planning, the majority of the current panel favoured a two-way classification.

Measurement techniques for tumour height vary widely, including rectilinear (one or more straight lines) and curvilinear measurements, either along the rectal wall or the centre of the rectal lumen. While statistical agreement between MRI and endoscopic measurements is generally good, discrepancies in tumour classification may arise, depending on the method used [21, 22]. The panel agreed that more uniform definitions would support more consistent multidisciplinary team (MDT) discussions and treatment guidance. Although full consensus was not reached, 77% supported measuring tumour height using one or more straight lines from either the anorectal junction or anal verge. The choice of landmark may depend on institutional preference, but must be clearly defined and applied consistently to avoid variations—such as those caused by including or excluding the anal canal, which typically adds 3–5 cm to the measurements.

### Staging low rectal cancers

A 2022 case-based survey by Lambregts et al identified staging of low rectal tumours as a key area of controversy, with low agreement (45–73%) for cT-categorisation among over 300 respondents [23]. Several classification systems have been proposed, mainly focused on (inter)sphincteric involvement to assess suitability for sphincter-preserving surgery, but none have been widely adopted into current guidelines. In the publication by Lambregts et al, a multidisciplinary expert panel proposed that radiological cT-categorisation should be based on the extent of tumour invasion at the level of the rectum and that involvement of the internal anal sphincter and intersphincteric space should not be taken into account. To align with pathology definitions that classify skeletal muscle invasion (e.g. external anal sphincter, puborectalis, levator ani) as pT4b disease, the panel from that study recommended that such involvement on MRI be staged as cT4b. Moreover, they suggested adding a suffix in the conclusion of the report to indicate anal canal involvement (i.e. “anal+”) [23]. The current panel reached consensus to adopt these definitions and reiterated the importance of clearly describing the specific layers involved and the craniocaudal extent of invasion to guide surgical planning.

### Definitions for MRF involvement

Consistent with previous guideline editions, a margin of ≤ 1 mm was confirmed as the criterion for MRF involvement. The intermediate category of ‘threatened MRF’ (1–2 mm) was abandoned to align with other major guidelines and to avoid confusion, supported by the MERCURY group data showing ≤ 1 mm as the most clinically relevant cut-off to identify patients at risk for local recurrence [24, 25]. The panel also refined the definition of MRF involvement: in addition to direct tumour extension, the MRF is considered involved when there is a ≤ 1 mm margin from either EMVI or irregular nodules (i.e. TDs or lymph nodes with extracapsular extension). This is based on evidence showing local recurrence risks of 20% (EMVI) and 31% (irregular nodes/TDs), compared to a 42% risk for direct tumour invasion [26]. Conversely, the MRF should be considered as non-involved in case of lymph nodes (regardless of size) with smooth margins within ≤ 1 mm from the MRF, as these have not been associated with increased recurrence risk [27, 28].

### Lymph nodes, TDs and EMVI

The 2018 guideline acknowledged the limitations of MRI for lymph node assessment but introduced a pragmatic framework combining size and morphology to guide clinical decision-making. These criteria aimed to establish more stringent norms to diagnose cN+ disease to reduce the risk of overstaging. Adoption of these criteria has contributed to improved specificity and a reduction in preoperative radiotherapy in the Netherlands [29].

While recent studies confirm a correlation between the 2018 ESGAR criteria and nodal malignancy, diagnostic accuracy remains suboptimal [30]. Niu et al compared the ESGAR criteria to Node-RADS (a three-level cumulative risk score of size and morphology). Node-RADS slightly outperformed ESGAR criteria (AUC 0.86 vs 0.80) to predict cN+ disease, and both clearly outperformed size alone, supporting the benefit of incorporating morphology [31]. However, morphological criteria may be less reproducible, especially in smaller-sized nodes [32]. In the study by Niu et al, the integration of ESGAR or Node-RADS with other clinical parameters (CEA, tumour location and size) led to further improved performance [31]. Reports from other groups confirm that the risk of node-positivity is associated with other risk factors such as more advanced cT-category, EMVI and a higher number of suspicious lymph nodes [33, 34]. Reflecting this, the panel supports a more patient-level and risk-adaptive evaluation, rather than a strict node-by-node analysis. Acknowledging the inherent uncertainties of lymph node assessment, the updated guidelines recommend including a confidence level when reporting, estimating the final cN-category as cN0, possibly cN+ or cN+ to better inform clinical decision making, as outlined in Fig. 3

In the 2018 guideline, no specific criteria were recommended for assessing lateral lymph nodes due to insufficient evidence. However, based on recent data from the Lateral Node Study Consortium and Dutch Snapshot Research Group [35–37], the panel now endorses a size cut-off of ≥ 7 mm to define malignant lymph nodes in the obturator and internal iliac regions. The panel also cautiously supports incorporating malignant morphological features to assess intermediate-sized nodes (5–7 mm), although evidence to support this remains limited [36]. Additionally, uniform definitions distinguishing regional vs non-regional lymph nodes and their implications for cN- and cM- categorisation have been integrated into the reporting template (Fig. 2*)*.

Regarding TDs, pathology data have clearly demonstrated that—although associated with the presence of lymph node metastases—they have significantly worse prognostic implications [38]. Moreover, TDs are strongly linked to the presence of EMVI [38, 39]. While TDs were included in the previous reporting template, no detailed guidance was provided on how to distinguish them from lymph nodes or incorporate them into TNM staging. Lord et al proposed definitions for TDs, describing them as nodules arising within/along venous channels, in continuity with major venous branches within the mesorectum and discontinuous from the main tumour. In contrast, lymph nodes were characterised by the familiar (oval) shape and capsule typical of lymph nodes [39]. Lv et al retrospectively tested these criteria in 130 patients, reporting an AUC of 0.77 but a low PPV (44%) [40]. Further validation is limited, although the ongoing COMET trial in the UK is currently investigating the reproducibility of these definitions, the radiologic-pathologic concordance, and prognostic value [41]. Meanwhile, criteria similar to those proposed by Lord et al (and including irregular shape) have been “provisionally” adopted by expert panels such as the Society of Abdominal Radiology colorectal and anal disease focus panel [8, 42]. Although our current guideline panel questions whether MRI can reliably differentiate lymph nodes from TDs, they adopted Lord et al’s criteria with 92% consensus, aiming to offer a pragmatic clinical approach. The panel further agreed that, for the time being, all suspicious nodules (whether regarded as lymph nodes or TDs) should be combined to determine the cN-category, in line with the current TNM 8^th^ edition [43]. However, most panel members question whether the N1c category (= TDs in the absence of nodal metastases) can be reliably assigned based on MRI. Given the strong association between TDs and EMVI, the two are often considered to be part of the same underlying mechanism of tumour dissemination [44]. Therefore, it may make more sense to group TDs with EMVI, or at least consider TDs as a separate entity from lymph nodes, considering that they do not share the same prognosis. As such, most of the panel agreed that a separate prose description detailing the (potential) presence of TDs should be included in the report, in addition to the cN-category.

### Use of DWI and other functional and quantitative imaging methods

DWI remains the only functional imaging technique recommended for routine use, primarily for qualitative (visual) assessment alongside T2W MRI and apparent diffusion coefficient (ADC) maps. Numerical ADC metrics or thresholds are not recommended, as current evidence does not support their use in clinical staging. DWI can aid tumour detection, particularly for smaller tumours or those obscured by faeces. However, the panel acknowledges that—based on recent evidence—DWI has a limited role in further staging, showing no significant added benefit for cT-category, EMVI, or MRF assessment [45–48]. As outlined in a review by Schurink et al, DWI may aid in the detection and localisation of lymph nodes, but its PPV for cN+ disease is low (± 50%), as both benign and malignant lymph nodes appear hyperintense, making DWI unsuitable for nodal characterisation [49].

In addition to the previous guidelines, the panel has now provided more detailed recommendations for DWI acquisition, including the use of reduced FOV DWI, which has been demonstrated to result in improved image quality with the potential to enhance diagnostic accuracy [50, 51]. Some panellists acknowledge, however, that reduced FOV acquisitions may exhibit artefacts, and full FOV sequences have the benefit of aiding in localising lymph nodes. Institutional preference may therefore guide protocol selection. DWI sequences should be acquired in the same orientation as the oblique-axial T2W sequences to allow direct comparison. Dynamic contrast-enhanced (DCE) MRI is not recommended for routine clinical staging.

While emerging Radiomics and AI models show promise for staging, tumour segmentation, and prognostication [52], the panel agreed that these tools are not yet ready for clinical use. Key concerns include small sample sizes, retrospective study designs, and a lack of prospective, externally validated evidence demonstrating its clinical benefit.

### Methodological limitations

Although all panellists completed the second questionnaire and voting round, two panellists did not complete the first questionnaire, and the results for round one were calculated based on the input of 24/26 panellists. Considering the high level of consensus that was reached after the second questionnaire round (and the logistical challenge in organising a face-to-face round with 26 panellists and three chairs), remaining areas of non-consensus were addressed during manuscript revision with feedback from all panellists.

### Conclusions and future outlook

These updated guidelines offer comprehensive recommendations for MRI acquisition, interpretation, and reporting to guide the clinical management of newly diagnosed rectal cancer. Key updates include refined criteria for tumour localisation, MRF involvement, and the assessment of lymph nodes and TDs. As these guidelines are adopted into practice, consistent widespread implementation and robust quality control will be essential to maximise their impact. This includes structured training and calibration initiatives to address both inter- and intra-observer variability, building on previous initiatives following the release of the 2018 guideline editions [53]. To support ongoing quality assurance, audit metrics should be developed to monitor adherence to newly introduced reporting elements, such as the STO and the confidence-level approach for cN-category assessment. Key indicators may include the proportion of reports incorporating these elements and their influence on treatment decisions.

In the coming years, a further shift is anticipated from traditional TNM-based staging toward more tailored, risk-adaptive approaches. Growing awareness and patient preference for organ-preserving treatments will drive the demand for more precise identification of early-stage tumours and better prediction of neoadjuvant treatment outcomes, especially in efforts to avoid radical surgery. While still considered premature for widespread clinical implementation, quantitative imaging techniques—such as tumour volumetry, advanced DWI metrics, and AI-based predictive models—are likely to become integral to clinical trials and validated for clinical use. AI-enabled acquisition and reconstruction techniques, such as deep learning accelerated T2W imaging, are also expected to be increasingly adopted into clinical protocols. Alongside these advancements, emerging biomarkers like circulating tumour DNA and genetic profiling markers (e.g. mismatch repair status) will enhance clinical decision-making. Timely updates to guidelines will be essential for incorporating these innovations into radiological practice.

## Supplementary information

ELECTRONIC SUPPLEMENTARY MATERIAL

## Data Availability

Not applicable (consensus guideline).

## Abbreviations

ADC: Apparent diffusion coefficient
cT: Clinical T-category
cN: Clinical N-category
cM: Clinical M-category
DCE: Dynamic contrast enhanced
DWI: Diffusion-weighted imaging
EMVI: Extramural venous/vascular invasion
ESGAR: European Society of Gastrointestinal and Abdominal Radiology
FOV: Field of view
MDT: Multidisciplinary team
MRF: Mesorectal fascia
STO: Sigmoid take-off
TD: Tumour deposit
TNM: Tumour nodes metastases
