# Supplementary material for: MRI to guide clinical management of rectal cancer: updated consensus recommendations from the European Society of Gastrointestinal and Abdominal Radiology (ESGAR)—PART I primary staging
Source: Eur Radiol. 2026 Jan 29;36(6):4592–607. doi: 10.1007/s00330-025-12274-w (PMC13212678; doi:10.1007/s00330-025-12274-w)
Supplement: Supplementary file 1 — ELECTRONIC SUPPLEMENTARY MATERIAL [file 330_2025_12274_MOESM1_ESM.pdf]

MRI to guide clinical management of rectal cancer: updated consensus recommendations from the European Society of Gastrointestinal and Abdominal Radiology (ESGAR) – PART I Primary Staging

ELECTRONIC SUPPLEMENTARY MATERIAL

Evidence synthesis & draft statements

Table of contents

I – Patient preparation 3
Overview of local practice of panelists 3
1. Use of spasmolytics and impact on (re)staging accuracy 3
2. Use of endorectal filling and impact on (re)staging accuracy 4
3. Use of a preparatory micro-enema and impact on (re)staging accuracy 4
II – MR Imaging protocol 5
T2-weighted MRI: 5
4. Recommended in plane resolution 5
5. Additional acquisition recommendations 5
DWI: 6
6. Benefit of applying more or higher b-values 6
7. Specific additional acquisition recommendations for DWI 6
III – Baseline staging (T2W) 7
Baseline tumour (T) staging 7
8. T2W MRI for baseline T-staging 7
9. T-staging in distal tumours involving the anal canal and/or pelvic floor 10
Baseline nodal (N) staging: 11
Mesorectal nodes: 11
10. T2W MRI for primary nodal staging 11
11. Nodal staging criteria from 2018 ESGAR guidelines 11
12. Evidence on alternative nodal staging criteria 11
13. Clinical relevance of establishing the total number of lymph nodes on MRI 11
Lateral nodes: 13

14. Criteria for lateral nodal staging 13
15. Definitions for regional (N) and non-regional (M) lymph node stations 13
Tumour deposits: 15
16. Criteria to discriminate between LNs and TDs 15
17. Performance of T2W MRI to discern LNs and TDs 15
18. Subclassification of tumour deposits based on their relation to adjacent vessels 15
Baseline EMVI assessment 16
19. Criteria for EMVI+ 16
20. Performance of T2W MRI 16
Baseline MRF assessment 18
21. MRF invasion (incl. invasion by tumour bearing structures other than the primary tumour) 18
IV – Restaging (T2W) 19
Response assessment and tumour (yT) restaging 19
22. T2W MRI for yT-restaging after CRT 19
23. mrTRG to assess a complete response 21
24. Other response grading systems (modified TRG, split scar, DWI patterns) 21
Nodal (yN) restaging 22
25. T2W MRI for yN-staging 22
26. Lateral nodal restaging after CRT 24
yEMVI assessment 25
27. Performance of T2W MRI for yEMVI assessment 25
28. Criteria to stage tumours as yEMVI+ after neoadjuvant treatment 25
yMRF assessment 26
29. Performance of T2W MRI for yMRF assessment 26
T2W Other 26
30. Criteria for restaging after non-standard neoadjuvant treatment 26
31. - Criteria to assess response in mucinous tumours - Prognostic significance of mucinous transformation in primarily non-mucinous tumours 27
32. How to incorporate fibrosis when assessing tumour height and length after CRT 28
33. Lexicon to classify tumours into different response groups after CRT 28
V – Diffusion-weighted imaging 29
Baseline staging 29
34. -39. Performance of DWI for T-, N-, EMVI and MRF and to discern LNs from tumour deposits? 29
Restaging 31
40. Performance of DWI (visual assessment) to assess a complete response vs residual tumour after neoadjuvant treatment 31
41. -44. Performance of DWI for yT-, yN-, yEMVI, yMRF staging 32
VI – Miscellaneous 34

45. Sigmoid take-off (STO) to discern rectal from sigmoid cancers 34

46. Benefit of classifying the degree of mucin 35

47-48. - How to classify rectal tumour location on MRI (e.g., as distal/mid/high or distal/high) - Use of the anorectal junction versus anal verge? - Are there additional methodological recommendations? 36

49. Use of Radiomics and other AI models for (re)staging 37

I – Patient preparation

Overview of local practice of panelists

**Summary of local practice of panelists (n=24 who completed surve /)**		
	**Primary staging**	**Restaging**
**Spasmolytics**	71%	71%
**Endorectal filling**	13%	13%
**Micro-enema**	38%	46%

1. Use of spasmolytics and impact on (re)staging accuracy

Draft statement 1:

Spasmolytics are optional, especially for upper rectal tumours or in patients with small bowel loops descending low in the pelvis in whom bowel movement artefacts are more likely to occur

(evidence level IV)

Summary of relevant literature:
One retrospective case control study (Taylor 2023; n=74) compared T-stage accuracy in 48 patients undergoing MRI with spasmolytics to 26 patients without spasmolytics; results showed no significant impact. One prospective study (Johnson 2007; n=47) showed significantly improved image quality and lesion characterization on pelvic MRI (not specifically rectum) with spasmolytics, but did not investigate its impact on staging accuracy. Further recommendations in literature are mainly based on narrative reviews or expert opinion with much variation in practice between centers (including our own panel, see above).

In summary, Intravenous (or intramuscular) administration of spasmolytics can improve image quality by reducing bowel movement artefacts, but there is no evidence that its use has a significant impact on (re)staging accuracy.

- Taylor A, et al. The effect of anti-spasmodic administration on the accuracy of magnetic resonance imaging staging of rectal cancer. ANZ J Surg 2023 Jun;93(6):1613-1619. doi: 10.1111/ans.18252. Epub 2023 Jan 4.

- Johnson W, et al. The value of hyoscine butylbromide in pelvic MRI. Clin Radiol. 2007 Nov;62(11):1087-93. doi: 0.1016/j.crad.2007.05.007.

2. Use of endorectal filling and impact on (re)staging accuracy

Draft statement 2:

Endorectal filling is not recommended

(evidence level II)

Summary of relevant literature:
One prospective, consecutive study (Ye 2016; n=50) compared lesion visualization, T- and N- stage accuracy on MRI before and after rectal distension (within the same patient cohort). No significant impact on staging accuracy was found, but lesion conspicuity improved. A second study (Stijns 2018; n=47) also compared staging accuracy with and without rectal filling and found no statistically significant effect. Several studies (Stijns 2018, Dal Lago 2005, Slater 2006) showed that rectal distension potentially results in compression of surrounding structures and affects the distance between the rectum and surrounding structures (in particular the MRF), though no hard evidence exists proving that this has a significant negative effect on staging accuracy incl. MRF assessment. Further recommendations in literature are mainly based on narrative reviews or expert opinion.

In summary, there is no evidence showing that endorectal filling has a significant positive effect on staging accuracy. Potential benefits include improved visualization and delineation of small tumours, and reduction of gas-induced susceptibility effects on DWI. Downsides include the impact of rectal filling on distance measurements to surrounding anatomic landmarks (in particular the MRF) and induction of T2 shine through effects on DWI.

- Ye F, et al. JOURNAL CLUB: Preoperative MRI Evaluation of Primary Rectal Cancer: Intrasubject Comparison With and Without Rectal Distention. AJR Am J Roentgenol 2016 Jul;207(1):32-9. doi: 10.2214/AJR.15.15383.
- Stijns RCH, et al. The influence of endorectal filling on rectal cancer staging with MRI. Br J Radiol 2018 Sep;91(1089):20180205.doi: 10.1259/bjr.20180205
- Dal Lago A, et al. Magnetic resonance imaging of the rectum during distension. Dis Colon Rectum. 2005 Jun;48(6):1220-7. doi: 10.1007/s10350-004-0933-0.
- Slater A, et al. Distance between the rectal wall and mesorectal fascia measured by MRI: Effect of rectal distension and implications for preoperative prediction of a tumour-free circumferential resection margin. Clin Radiol 2006 Jan;61(1):65-70. doi:10.1016/j.crad.2005.08.010.

3. Use of a preparatory micro-enema and impact on (re)staging accuracy

Draft statements 3 & 4:

A preparatory micro-enema is recommended for restaging to reduce susceptibility artefacts on DWI

(evidence level IV)

A preparatory micro-enema is optional for baseline staging

(evidence level III-IV)

Summary of relevant literature:
Two case control studies (Jayaprkasam 2021; n=507, van Griethuysen 2018; n=335) reported significantly less artefacts on DWI post-therapy in patients who received a preparatory micro-enema compared to patients who were scanned without; impact on diagnostic performance was not assessed in either of these studies. Jayaprkasam also evaluated 191 baseline MRIs and found no effect of a micro-enema on artefacts in this setting.

A single study (Viktil; n=73) suggested that at baseline a micro-enema may be beneficial to stage early cancers (up to T1sm2) due to the beneficial effect of submucosal edema caused by the microenema, but no comparison was provided with scans performed without a micro-enema.

Further recommendations in literature are mainly based on narrative reviews or expert opinion with much variation in practice.

In summary, there is some evidence showing that a micro-enema reduces artefacts on DWI (which is typically mainly used in the restaging setting), but there is limited evidence suggesting a beneficial effect for baseline staging.

- Jayaprakasam VS, et al. Does microenema administration improve the quality of DWI sequences in rectal MRI? Abdom Radiol (NY). 2021 Mar;46(3):858-866. doi: 10.1007/s00261-020-02718-w.
- van Griethuysen JJM, et al. Gas-induced susceptibility artefacts on diffusion-weighted MRI of the rectum at 1.5 T - Effect of applying a micro-enema to improve image quality. Eur J Radiol. 2018 Feb;99:131-137. doi: 10.1016/j.ejrad.2017.12.020.

II – MR Imaging protocol

T2-weighted MRI:

4. Recommended in plane resolution

5. Additional acquisition recommendations

Draft statements 5 & 6:

In plane resolution for T2W MRI should be less than 1 x 1 mm

(evidence level V)

The protocol should include at least one large FOV T2W (or alternatively T1W) sequence covering all pelvic compartments from the level of the aortic bifurcation / promontory to the distal margin of the anal canal including the inguinal lymph node regions; this sequence may

either be acquired as part of the high resolution protocol or added as a fast-acquisition sequence (e.g. SSFSE, HASTE)

(evidence level V)

Summary of relevant literature:
There are no studies that have compared diagnostic performance for different T2W spatial resolutions. Publications from Australia (Gormly 2021; Australian guidelines) have adopted a recommended in plane resolution of 0.6 x 0.6 mm (defined as high-resolution), based on definitions used in the MERCURY studies. Expert opinion-based reviews (Miranda 2023; Gourtsoyianni 2016, Fraum 2023) recommend an in plane resolution of less than 1 x 1 mm. Limited further protocol recommendations are provided in different available radiological guidelines, though most expert reviews include recommendations on the required FOV, as outlined in the draft statement.

- Gormly K. Rectal MRI: the importance of high resolution T2 technique. Abdominal Radiology (2021) 46:4090–4095. https://doi.org/10.1007/s00261-021-03047-2
- Miranda et al. Advances in MRI-Based Assessment of Rectal Cancer Post-Neoadjuvant Therapy: A Comprehensive Review. J Clin Med 2023 Dec 28;13(1):172. doi: 10.3390/jcm13010172
- Gourtsoyianni S, Papanikolaou N. Role of Magnetic Resonance Imaging in Primary Rectal Cancer—Standard Protocol and Beyond. Semin Ultrasound CT MRI 2016; 37:323-330
- Fraum et al. The optimized rectal cancer MRI protocol: choosing the right sequences, sequence parameters, and preparatory strategies. Abdominal Radiology (2023) 48:2771–2791.

DWI:

6. Benefit of applying more or higher b-values

7. Specific additional acquisition recommendations for DWI

Draft statements 7 & 8:

For tumour response assessment, DWI should be acquired in the same plane as the axial T2weighted sequence (oblique-axial, perpendicular to the tumour axis)

(evidence level V)

For tumour response assessment, reduced FOV DWI is recommended; full FOV acquisitions are optional to ensure complete assessment of the whole pelvis including all pelvic nodal stations

(evidence level III)

Summary of relevant literature:
Two studies (Bates 2020; n=28, Delli Pizzi 2019; n=55) compared ultrahigh b-values (b1500-2000) to standard b800-b1000 DWI. No significant differences in staging accuracy were found; Delli Pizzi did show improved lesion conspicuity and IOA.

Two studies (Peng 2018; n=81, Jang 2021; n=73) compared reduced FOV DWI to full FOV DWI within the same patient cohort and showed significantly improved image quality for the reduced FOV sequence. Jang also showed a positive effect for local tumour response assessment after CRT with significantly higher diagnostic accuracy for two readers to assess a complete response using the reduced FOV sequence (72-78% versus 57-61% for the full FOV sequence)

Some incidental reports have shown good results for technical developments (e.g., computed high bvalue images, multislice-accelerated DWI, readout-segmented DWI, tilted excitation plane DWI, integrated dynamic shimming, and deep learning reconstruction techniques). However, these techniques remain to be validated and to date no strong evidence base exists to support their routine use.

<u>In summary</u>, the panel has previously already agreed that a DWI sequence should include at least 1 high b-value of b800 or higher. There is no evidence suggesting a significant benefit for applying multiple b-values or ultra-high b-values (>b1500-2000). With respect to further acquisition parameters, reduced FOV DWI appears to offer improved diagnostic quality and accuracy to assess local tumour response compared to full FOV DWI; full FOV acquisitions may be used (as an add on) to ensure complete assessment of the whole pelvis including all pelvic nodal stations. Though there is no specific evidence to support this, expert opinion suggests to acquire DWI in the same plane as the axial T2-weighted sequence (oblique-axial, perpendicular to the tumour axis).

- Bates DV, et al. Diagnostic accuracy of b800 and b1500 DWI-MRI of the pelvis to detect residual rectal adenocarcinoma: a multi-reader study. Abdom Radiol (NY). 2020 Feb;45(2):293-300. doi: 10.1007/s00261-019-02283-x.
- Delli Pizzi A, et al. Tumour detectability and conspicuity comparison of standard b1000 and ultrahigh b2000 difusion-weighted imaging in rectal cancer. Abdom Radiol (NY) 2019 Nov;44(11):3595-3605. doi: 10.1007/s00261-019-02177-y.
- Jang S, et al. Reduced field-of-view versus full field-of-view difusion-weighted imaging for the evaluation of complete response to neoadjuvant chemoradiotherapy in patients with locally advanced rectal cancer. Abdom Radiol (NY) 2021 Apr;46(4):1468-1477. doi: 10.1007/s00261-020-02763-5 - Peng Y, et al. Comparison of Reduced Field-of-View Diffusion-Weighted Imaging (DWI) and Conventional DWI Techniques in the Assessment of Rectal Carcinoma at 3.0T: Image Quality and Histological T Staging. J Magn Reson Imaging 2018 Apr;47(4):967-975. doi: 10.1002/jmri.25814.

III – Baseline staging (T2W)

Baseline tumour (T) staging

8. T2W MRI for baseline T-staging

Draft statement 9

MRI can be used to distinguish low-risk from high-risk T-stage by subclassifying tumours as ≤cT3ab versus >≥ycT3cd

(level of evidence I)

Summary of relevant literature:

Several meta-analyses (and individual reports) have assessed accuracy for T2W T-staging. MRI has the highest sensitivity to detect cT3-4 tumours; sensitivity to detect T1-T2 tumours is relatively low (around 45%).

Several studies have shown that ERUS outperforms MRI in detecting T1 lesions; staging of T1 tumours remains challenging as shown by a recent meta-analysis (Luglio 2022; n=331 patients from 7 studies) who reported a pooled sensitivity of 0.47 for MRI to identify T1 tumours (versus 0.78 for ERUS).

Two groups have tried to improve the staging of early rectal cancer with high-resolution MRI and to identify lesions eligible for local excision (i.e. T1sm1-2 vs. T2sm2-T3ab) and found encouraging results. Viktil et al. (n=50) showed that by using a bisacodyl containing micro-enema the submucosa widened and this led to a better estimation of the invasion depth of the lesion. However, these results have not yet been reproduced by other groups.

Taylor et al (2011; n=374) showed that MRI can select good prognosis tumours with a low (3%) risk for local recurrence when managed with surgery only by classifying them as ≤ cT3ab, MRF- and any N.

With respect to T4 stage, Sim et al (2021; n=55) showed that MRI has moderate accuracy to detect
T4a tumours with a sensitivity of 69% and specificity of 76% (though the reference standard in this study was intraoperative findings and not histology). A second study by Alvarez Sarrado et al. (2022; n=161) that did correlate MRI findings with histopathology, showed an accuracy of 81% (sensitivity 64%; specificity 87%) for identifying peritoneal reflection invasion (=T4a). IOA was 0.51. Based on available evidence, MRI is more accurate in staging cT4b than cT4a disease. Main problem is overstaging of cT4a (PPV 47%).

In summary, T2-weighted MRI has moderate accuracy for T-staging. Highest accuracy is achieved for cT3-4 tumours; performance (in particular sensitivity) to identify T1 tumours is lowest. The panel previously agreed that MRI is the recommended primary staging modality for all T-stages, except for the differentiation and staging of T1 tumours for which ERUS is the preferred modality.

Overall T-staging

		T1		T2		T3		T4	
Studie	N=	sens	spec	sens	spec	Sens	spec	sens	spec
Zhang2016 (meta-analysis)	858	58%	97%	62%	81%	80%	74%	71%	97%
Detering2020	5539	45%	93%	92%	26%	-	-	-	-
Beets-Tan2001	76	-	-	38-46%	83-94%	83-95%	61-75%	75-100%	100
Luglio 2022 (meta-analysis)	331	47%	98%	61%	86%	81%	77%	75%	95%

Specific low versus high-risk subgroups:

Studie	N=	sens	spec
≥T2 (positive outcome) versus ≤T1			
Bipat2004 (meta-analysis)	90 studies	94%	69%

≥T3 (positive outcome) versus ≤T2			
Bipat2004 (meta-analysis)	90 studies	82%	76%
Al-Sukhni 2012	19 studies	87%	75%
Balyasnikova 2017	65	91%	85%
≥T4 versus ≤T3			
Bipat2004 (meta-analysis)	90 studies	74%	96%

Early tumours:

Study	Outcome	N=	sens	spec
Viktil2024	T1 sm1-2 versus T1sm3-T3ab	73	86-93%	83-90%
Balyasnikova2017	T1 sm1-2 versus T1sm3-T3ab	65	93%	48%

- Luglio et al. Endorectal Ultrasonography and Pelvic Magnetic Resonance Imaging Show Similar Diagnostic Accuracy in Local Staging of Rectal Cancer: An Update Systematic Review and Meta-Analysis. Diagnostics 2021;12(1), 5: doi **10.3390/diagnostics12010005**.
- Viktil et al. MRI of early rectal cancer; bisacodyl micro-enema increases submucosal width, reader confidence, and tumour conspicuity. Abd Radiol 2024; https://doi.org/10.1007/s00261-024-04701-1
- Al-Sukhni et al. Diagnostic accuracy of MRI for assessment of T catgory, Lymph node metastases and circumferential resection margin involvement in patients with rectal cancer: a systematic review and metaanalysis.. Ann Surg Oncol 2012; 19: 2212-2223.
- Balyasnikova et al. Diagnostic accuracy of high-resolution MRI as a method to predict potentially safe endoscopic and surgical planes in patients with early rectal cancer. BMJ Open Gastroenterology 2017;4:e000151. doi: 10.1136/bmjgast-2017-000151
- Bipat et al. Rectal cancer: local staging and assessment of lymph node involvement with endoluminal US, CT,and MR imaging: a meta-analysis. Radiology 2004; 232: 773-83 doi.org/10.1148/radiol.2323031368
- Beets-Tan et al. Accuracy of magnetic resonance imaging in prediction of tumour-free resection margin in rectal cancer. Lancet 2001; 357:497-504.
- Detering et al. MRI cT1-2 rectal cancer staging accuracy: a population-based study. Br J Surg 2020;107:13721382.
- Zhang et al. Diagnostic accuracy of MRI for assessment of T category and circumferential resection margin involvement in patients with rectal cancer: a meta-analysis. Dis Colon Rectum 2016;59:189-99.
- Taylor et al. Preoperative high-resolution magnetic resonance imaging can identify good prognosis stage 1, 2 and 3 rectal cancer best managed by surgery alone: a prospective multicenter, European study. Ann Surg 2011;253:711-9.
- Sim et al. Accuracy of MRI for prediction anterior peritoneal reflection involvement in locally advanced rectal cancer: a comparison with intraoperative findings. Abd Radiol 2022; 47: 508-516.
- Alvarez-Sarrado et al. Rectal cancer at the peritoneal reflection. Preoperative MRI accuracy and histopathologic correlation. Prospective study. Cir Esp 2022; 100(8): 488-495.

9. T-staging in distal tumours involving the anal canal and/or pelvic floor

Draft statements 10, 11 and 12:

T staging should primarily be informed by the extent of tumour invasion at the level of the rectum; involvement of the internal anal sphincter and intersphincteric space should not be taken into account in T-stage categorization.

(evidence level V)

Invasion of the pelvic floor (puborectalis, levator ani) muscles should be staged as T4b

(evidence level V)

Involvement of the external anal sphincter should be staged as T4b

(evidence level V)

Summary of relevant literature:

A recent case-based survey among 255 radiologists and 66 other clinicians (Lambregts 2022) identified T-staging in low rectal cancers involving the anal canal as one of the key areas of controversy in radiological TNM staging with low agreement (45-73%) among survey respondents.

There is no evidence on how invasion into different layers of the anal canal (and pelvic floor) translates into long-term patient outcomes. It has mainly been shown that invasion of the intersphincteric plane and external sphincter is associated with a higher risk of positive resection margins when performing TME (Salerno 2009). Several classification systems to address low rectal cancer have been proposed, mainly driven by surgical implications (whether or not sphincterpreserving surgery will be feasible), but none have been widely adopted into current guidelines. In the survey paper by Lambregts, a multidisciplinary expert panel (incl. radiologists, surgeons, pathologists and radiation oncologists) proposed that radiological T staging should primarily be informed by the extent of tumour invasion at the level of the rectum and that involvement of the internal anal sphincter and intersphincteric space should not be taken into account in T-stage categorization. Considering that pathologists consider skeletal muscle invasion as pT4b disease and aiming to avoid inconsistencies between radiology and pathology reports, the panel furthermore recommended that involvement of the external anal sphincter, puborectalis, or levator ani muscles (i.e., skeletal muscles) should be classified as cT4b. The disease focus panel from SAR (Lee 2023) did not reach consensus whether external anal sphincter involvement should be classified as T4b, but also agreed that in reporting anal involvement, a specific description of the level of involvement, and the location/length (upper/mid/distal) of the involvement should be communicated to the surgeon, to help select the appropriate surgical option.

In summary, there seems to be a tendency in literature to primarily base radiological T-stage on the depth of invasion at the level of the rectum and to not include involvement of the internal sphincter or intersphincteric plane in T-stage categorization. Pelvic floor invasion is generally considered by experts as T4b disease. There is no clear consensus in literature (among radiologists) if external sphincter invasion should be considered T4b or discarded from T-stage categorization, though pathologists generally consider it as T4b.

- Lambregts et al. Current controversies in TNM for the radiological staging of rectal cancer and how to deal with them: results of a global online survey and multidisciplinary expert consensus. Eur Radiol 2022;32(7):4991-5003.
- Salerno et al. Magnetic Resonance Imaging Prediction of an Involved Surgical Resection Margin in Low Rectal Cancer. Dis Colon Rectum 2009; 52: 632Y639
- Lee et al. Rectal cancer lexicon 2023 revised and updated consensus statement from the Society of Abdominal Radiology Colorectal and Anal Cancer Disease-Focused Panel. Abdominal Radiology (2023) 48:2792–2806

Baseline nodal (N) staging:

Mesorectal nodes:

10. T2W MRI for primary nodal staging

11. Nodal staging criteria from 2018 ESGAR guidelines

12. Evidence on alternative nodal staging criteria

13. Clinical relevance of establishing the total number of lymph nodes on MRI

Draft statements 13, 14 and 15:

Nodal stage should be reported as N0 or N+ and include a level of confidence (definitely N0; possibly N+; definitely N+)

(evidence level V)

Known risk factors associated with N+ disease (e.g. higher T-stage, presence of EMVI, higher number of nodes) should be taken into account when determining the risk for N+ stage

(evidence level V)

The nodal staging criteria proposed by ESGAR in 2018 (that combine size and morphology) are still recommended to evaluate individual nodes

(evidence level V)

Location and size of the most suspicious node(s) should be included in the report

(evidence level V)

Summary of relevant literature:
A meta-analysis (Zhuang 2021) compared different size cut-offs and morphology criteria for nodal staging. Sensitivities ranged from 62-81% and specificities from 64-91%. No clear criteria could be advised. AUC was 0.78 for the pooled performance of all approaches. Addition of morphological criteria improved diagnostic performance (albeit not significantly).

Niu et al (2024; n=154) compared the ESGAR criteria to Node-RADS and size-alone to predict pN0 versus pN+ stage. Node-RADS (a cumulative score of size and morphology) slightly outperformed ESGAR criteria (AUC 0.86 versus 0.80) but both clearly outperformed size only, supporting the benefit of combining size with morphology. Integration of ESGAR or Node-RADS with other clinical parameters (CEA, tumour location and size) led to a further

improved performance. Other reports (e.g., Haak 2021; Koh 2008) have also shown that the risk for node-positivity is associated with other risk factors such as higher T-stage and EMVI.

The ESGAR criteria were introduced to increase the threshold to (over)call nodes as positive and reduce the risk of overstaging. Adoption of these same criteria in the Dutch national guidelines has contributed to improved specificity for nodal staging and a reduction in the use of preoperative radiotherapy in the Netherlands as more patient are considered early-stage (Detering 2020; n=19.421). However, overstaging is still frequently encountered. Therefore, experts consider it advisable to – when in doubt – opt for the lowest N-stage category. This follows the basic staging principles of the TNM-staging system.

El Khababi et al (2023; n=75) showed considerably better IOA (among 21 radiologists) when nodal stage was reported dichotomous (N0/N+) versus a 3-way categorization (N0/1/2). Agreement to define the absolute number of suspicious lymph nodes was very poor (α=0.05). Staging performance was significantly influenced by the level of confidence with which the study readers assigned their Nstage. The authors therefore concluded that for staging factors with low reproducibility (such as Nstage), a level of confidence should be embedded into the report to better guide clinical decisions.

With respect to the overall number of mesorectal lymph nodes, a meta-analysis (Karjol 2020) has shown that lymph node harvest and the lymph node ratio at pathology (number of malignant nodes relative to the total number of harvested nodes) are both predictors of OS and DFS. The recommended number of harvested nodes is 12 or more; resulting in a low risk of pN-understaging. The clinical relevance of establishing the total number of lymph nodes on MRI has not been addressed in published literature. Unpublished data from the Netherlands Cancer Institute does show that, overall, readers are significantly more likely to assign a positive N-stage on MRI if the total number of visible nodes is higher. However, IOA to establish the absolute number of total lymph nodes is low.

In summary, MRI has moderate performance to stage mesorectal nodes. Combining size and morphology offers the most reliable results, but overstaging remains an issue and should be avoided. Performance to assign individual N-stages (N0/1/2 or even further subcategories) will lead to less accurate and reproducible results. As such, it may be better to assign a dichotomous N-stage (N0 or N+) accompanied by a level of confidence and description of the most suspicious node(s). The level of confidence should be determined taking into account the overall number of (potentially suspicious) lymph nodes, the size and morphology of the most suspicious node(s), as well as other risk factors associated with an increased risk for N+ disease.

- Niu et al. Diagnostic performance of Node-RADS score for mesorectal lymph node metastasis in rectal cancer. Abdom Radiol (NY). 2024 Jul 24. doi: 10.1007/s00261-024-04497-0.
- Zhuang et al. Magnetic resonance imaging evaluation of the accuracy of various lymph node staging criteria in rectal cancer: a systematic review and meta-analysis. Front Oncol 2021; 11: doi:
10.3389/fonc.2021.709070
- Haak et al. Prevalence of nodal involvement in rectal cancer after chemoradiotherapy. Br J Surg 2021;108:1251-1258. doi: 10.1093/bjs/znab194.
- Koh et al. The relationship between MR demonstration of extramural venous invasion and nodal disease in rectal cancer. Clin Med Oncol 2008;2:267-273
- Detering et al. Nationwide analysis of hospital variation in preoperative radiotherapy use for rectal cancer following guideline revision. Eur J Surg Oncol 2020;46:486-494
- El Khababi et al. Pearls and pitfalls of structured staging and reporting of rectal cancer on MRI: an international multireader study. Br J Radiol 2023;96(1150):20230091

- Karjol et al. Lymph Node Ratio as a Prognostic Marker in Rectal Cancer Survival: A Systematic Review and Meta-Analysis. Cureus 2020 10; 12(5).

Lateral nodes:

14. Criteria for lateral nodal staging

15. Definitions for regional (N) and non-regional (M) lymph node stations

Draft statements 16, 17, 18 and 19:

A size threshold of ≥ 7 mm (short-axis diameter) is recommended to diagnose malignant nodes in the obturator and internal iliac compartments

(evidence level II)

Presence of malignant morphologic features (round shape, loss of fatty hilum, internal heterogeneity and border irregularity) supports the suspicion of malignant lateral nodes in intermediate-sized nodes (5-7 mm)

(evidence level II)

Elongated nodes immediately dorsal to the external iliac veins should be considered benign

(evidence level II)

- N-stage lymph nodes include the mesorectal, obturator and internal iliac nodes
- M-stage lymph nodes include the external iliac and common iliac nodes
- Inguinal lymph nodes are considered M-stage nodes, except in tumours involving the anal canal below the dentate line, in which case they are still considered N-stage nodes

(evidence level V)

Summary of relevant literature:

The lateral node study consortium (Ogura 2019; n=1216) proposed a size threshold of ≥7 mm (short axis) for internal iliac and obturator lymph nodes as this is associated with a significantly higher risk (>20% which was arbitrarily defined as a "too high risk") for lateral nodal recurrence. Other features (signal heterogeneity, border contour) were not associated with distant recurrence or cancerspecific survival.

Two more recent papers based on the same Dutch population cohort data (Sluckin 2024; van Geffen 2024; n=3057 in total) both analyzed a selection of 284 patients who had visible lateral LNs (> 5 mm) at baseline. Sluckin reported that nodes ≥7 mm resulted in an increased risk (15%) for lateral local recurrence and that this risk increased further if malignant nodal features were present (though these features itself were not further specified). Van Geffen zoomed in further on the value of size and number of nodes, as well as the presence of malignant features and showed that the risk for lateral local recurrence was highest in patients with multiple enlarged (≥ 7 mm) nodes and enlarged nodes multiple malignant features (risk 17-28%). Nodes between 5-7 mm that showed more than one malignant feature also had an increased risk for lateral recurrence (8-13%) especially if

malignant features persisted after neoadjuvant treatment. Malignant features were defined as round shape, loss of fatty hilum, internal heterogeneity and border irregularity.

Ogawa et al (2016; n=449) reported a size threshold of 5 mm to be superior to 10 mm as a cutoff for lateral nodes, but more in-depth analyses incl. correlation with morphology were not provided. Other published reports concern smaller retrospective cohorts of 57-192 patients.

Elongated nodes immediately dorsal to the external iliac veins were considered benign in the reports by the lateral node study consortium as well as the Dutch studies; the report by Ogura et al. furthermore showed that enlarged external iliac nodes were not associated with a risk for lateral nodal recurrence. As shown in previous Japanese studies (Kanemitsu 2017; Yokoyama 2014) pathologic external iliac nodes are mainly associated with an increased risk for distant metastases and are therefore typically considered non-regional (M-stage) nodes.

A multidisciplinary expert consensus panel (Lambregts 2022) agreed to consider suspicious external iliac nodes (as well as common iliac nodes) as M+ disease, while suspicious obturator and internal iliac nodes are considered N+. Suspicious inguinal nodes are generally considered as M+, except in tumours involving the anal canal below the dentate line, in which case inguinal nodes are still considered as part of the N-stage.

In summary, the largest available studies to date support a size threshold ≥7 mm to diagnose obturator and internal iliac nodes as malignant at the time of baseline staging. Results as to whether malignant features (shape, border, signal) have significant prognostic implications are contradictory but most recent data support their benefit.

- Ogura et al. Neoadjuvant (Chemo)radiotherapy With Total Mesorectal Excision Only Is Not Sufficient to Prevent Lateral Local Recurrence in Enlarged Nodes: Results of the Multicenter Lateral Node Study of Patients With Low cT3/4 Rectal Cancer. J Clin Oncol. 2019 Jan 1;37(1):33-43. doi: 10.1200/JCO.18.00032.
- Ogura et al. Lateral Nodal Features on Restaging Magnetic Resonance Imaging Associated With Lateral Local Recurrence in Low Rectal Cancer After Neoadjuvant Chemoradiotherapy or Radiotherapy. JAMA Surg. 2019 Sep 1;154(9):e192172. doi: 10.1001/jamasurg.2019.2172.
- Sluckin et al. Prognostic Implications of Lateral Lymph Nodes in Rectal Cancer: A Population-Based Cross-sectional Study With Standardized Radiological Evaluation After Dedicated Training. Dis Colon Rectum 2024;67:42-53
- Van Geffen et al. Value of Size and Malignant Features of Lateral Lymph Nodes in Risk Stratification at Lateral Local Recurrence of Rectal Cancer: A National Cohort Study. J Natl Compr Canc Netw. 2024 Feb;22(1):17-25. doi: 10.6004/jnccn.2023.7081.
- Ogawa et al. Selection of Lymph Node-Positive Cases Based on Perirectal and Lateral Pelvic Lymph Nodes Using Magnetic Resonance Imaging: Study of the Japanese Society for Cancer of the Colon and Rectum. Ann Surg Oncol. 2016 Apr;23(4):1187-94. doi: 10.1245/s10434-015-5021-2.
- Kanemitsu et al. Potential impact of lateral lymph node dissection (LLND) for low rectal cancer on prognoses and local control: a comparison of 2 high-volume centers in Japan that employ different policies concerning LLND. Surgery. 2017;162(2):303-314. doi:10.1016/j.surg.2017.02.005
- Yokoyama et al. Survival benefit of lateral lymph node dissection according to the region of involvement and the number of lateral lymph nodes involved. Surg Today. 2014;44(6):1097-1103. doi:10.1007/s00595-013-0815-y
- Lambregts et al. Current controversies in TNM for the radiological staging of rectal cancer and how to deal with them: results of a global online survey and multidisciplinary expert consensus. Eur Radiol 2022;32(7):4991-5003.

Tumour deposits:

16. Criteria to discriminate between LNs and TDs

17. Performance of T2W MRI to discern LNs and TDs

18. Subclassification of tumour deposits based on their relation to adjacent vessels

Draft statements 20, 21, 22 and 23:

Recommended criteria to diagnose mesorectal nodules as tumour deposits and discern them from lymph nodes include irregular shape and contiguity with veins

(evidence level II-III)

All suspicious nodules (whether regarded as lymph nodes or tumour deposits) should be combined to determine the N-stage

(evidence level V)

N1c stage (presence of only tumour deposits in absence of any malignant nodes) cannot be reliably established on MRI

(evidence level V)

A prose description of the suspected presence of tumour deposits should be included in the report and conclusion

(evidence level V)

Summary of relevant literature:

A meta-analysis of pathology data (Nagtegaal 2017) showed that even though tumour deposits (TDs) are correlated with the presence of lymph node metastases and EMVI, they have worse prognostic implications. Lord et al. have proposed definitions to discriminate tumour deposits from (metastatic) lymph nodes and describe tumour deposits as nodules arising within/along venous channels, in continuity with major venous branches within the mesorectum and discontinuous from the main tumour, while lymph nodes are characterized by the familiar shape and capsule typical of lymph nodes. Lv et al (2023) retrospectively tested these criteria in 130 patients (including a mix of patients with/without neoadjuvant treatment) and found an AUC of 0.77, sens 74%, spec 79%, PPV 44%, NPV 94% and accuracy 79% to diagnose TDs. Further evidence on the performance of MRI (using these criteria) to distinguish between TDs and lymph nodes is limited; the COMET trial that investigates the reproducibility of these definitions and the concordance between MRI and histopathology, along with the prognostic implications is still ongoing in the UK. Meanwhile criteria similar to those proposed by Lord as well as irregular shape have been “provisionally” adopted by expert panels such as the SAR DFD (Lee 2023; Kassam 2022) to discern TDs, though multidisciplinary expert panel recommendations published by Lambregts (2022) were more cautious.

In a small study of n=30 Xu et al (2023) compared morphologic features as well as quantitative imaging features (derived from DWI and DCE) between lymph nodes and TDs.

They confirmed that irregular shape and border were more common in TDs and also showed found potential value in measuring DCE and DWI characteristics of nodules as well as the primary tumour to predict TD. However, these results have not been validated on larger scale.

With respect to the differentiation of TDs based on their relation to vessels, a pathology study by Ueno et al (2007) in 1027 CRC specimens (incl. 205 with TDs) showed that TDs of a “vascular invasion” type had a more favourable prognosis than non-vascular TDs (hazard ratio 2.5 versus 4.7). There is no evidence in literature focusing on whether a sub classification for radiologic staging of TDs according to the relation to vessels has prognostic or therapeutic implications.

In summary, solid evidence on the performance of MRI to discern tumour deposits from (metastatic) lymph nodes is lacking and by extension it is unclear whether MRI is able to establish an N1c stage. Preliminary data suggest that contiguity with veins and irregular shape are features that favour the diagnosis of TDs.

- Nagtegaal et al. Tumour deposits in colorectal cancer: improving the value of modern staging-a systematic review and meta-analysis. J Clin Oncol 2017 35(10):1119–1127
- Lord et al. Can extranodal tumour deposits be diagnosed on MRI? Protocol for a multicentre clinical trial. BMJ Open 2020;10(10): e033395. doi: 10.1136/bmjopen-2019-033395
- Lv et al. Identifying Tumour Deposits in Patients with Locally Advanced Rectal Cancer: using Multiplanar High-Resolution T2WI. Curr Med Imaging 2023; doi: 10.2174/1573405620666230825113550
- Lee et al. Rectal cancer lexicon 2023 revised and updated consensus statement from the Society of Abdominal Radiology Colorectal and Anal Cancer Disease-Focused Panel. Abdominal Radiology (2023) 48:2792–2806
- Kassam et al. Update to the structured MRI report for primary staging of rectal cancer. Abdominal Radiology (2022) 47:3364–3374
- Lambregts et al. Current controversies in TNM for the radiological staging of rectal cancer and how to deal with them: results of a global online survey and multidisciplinary expert consensus. Eur Radiol 2022;32(7):4991-5003.
- Xu et al. Distinguishing mesorectal tumour deposits from metastatic lymph nodes by using diffusionweighted and dynamic contrast-enhanced magnetic resonance imaging in rectal cancer. European Radiology (2023) 33:4127–4137
- Ueno et al. Extramural Cancer Deposits Without Nodal Structure in Colorectal Cancer Optimal Categorization for Prognostic Staging. Am J Clin Path 2007;127(2):287-94

Baseline EMVI assessment

19. Criteria for EMVI+

20. Performance of T2W MRI

Draft statement 24:

Grade 3 and grade 4 EMVI should be regarded as EMVI positive

(evidence level I)

Summary of relevant literature:

Most evidence on EMVI at primary staging is based on prognostic studies, as these patients tend to undergo CRT (rather than direct surgery) and will therefore not have direct rad-path correlation available. Several meta-analyses have evaluated the prognostic value of MRI-detected EMVI. Chen et al. (2021) performed a meta-analysis of n=2237 patients (both primary staging and restaging postneoadjuvant therapy) from 11 studies and found mrEMVI to predict DFS and OS with HRs ranging from 1.93-2.5 (results based on 8 studies). Similar findings were found by Tan et al. (2021), and Rouleau-Fournier et al (2022). The latter also found mrEMVI+ to be associated with a higher risk for synchronous metastasis.

How to grade EMVI on MRI was first reported by Smith et al (2008; n=142) who showed that a 5point grading system was useful for prognostication. EMVI assessment was correlated with histology with a sensitivity of 62% and specificity of 88% (in n=94 undergoing primary surgery). Recurrencefree survival was significantly less for patients with mrEMVI grade 3-4 vs grade 0-2 (35% vs. 74%). EMVI grade 3-4 (3=tumour signal extending into vessel with preserved vessel contour, 4=tumour signal extending into vessel with irregular countour or nodular expansion) was therefore defined as EMVI+ disease.

A recent study (van Geffen 2024; n=1213) evaluated different grades of EMVI and its impact on longterm outcome. Both grade 3 and grade 4 EMVI were significant predictors of impaired outcome (LR, DM, DFS, OS) compared to EMVI-negative (grade 0-2) patients. Grade 4 EMVI showed higher LRrates (21 vs 18%) and distant metastases (49 vs 30%) compared to grade 3 EMVI. In multivariable analysis hazard ratios to predict LR were however similar for grade 3 and 4 EMVI (HR 1.8 and 1.7, p=0.047). There is to date no evidence to establish whether the differentiation between grade 3 and 4 EMVI on MRI is reliable and reproducible. The study by van Geffen did not include any IOA analyses.

In summary, there is compelling evidence that mr-detected EMVI has prognostic significance. EMVI grade 3-4 is typically considered as EMVI positive disease. There is some evidence suggesting an added benefit for the separate reporting of grade 4 EMVI as a significantly worse prognostic factor, but it is unclear if this differentiation can accurately (and reproducibly) be made on MRI.

- Chen et al. The prognostic value of MRI-detected extramural vascular invasion (mrEMVI) for rectal cancer patients treated with neoadjuvant therapy: a meta-analysis. Eur Radiol 2021;31:8827-8837.
- Tan et al. Prognostic Importance of MRI-Detected Extramural Venous Invasion in Rectal Cancer: A Literature Review and Systematic Meta-Analysis. Int J radiat Oncol Biol Phys 2021;111:385-394.
- Rouleau Fournier, et al. Oncologic Outcomes Associated With MRI-detected Extramural Venous Invasion (mrEMVI) in Rectal Cancer: A Systematic Review and Meta-analysis. Ann Surg 2022;275:303314.
- Smith et al. Prognostic significance of magnetic resonance imaging-detected extramural vascular invasion in rectal cancer. Br J Surg 2008;95:229-236.
- Van Geffen et al. Prognostic significance of MRI-detected extramural venous invasion according to grade and response to neo-adjuvant treatment in locally advanced rectal cancer: a national cohort study after radiologic training and reassessment. Eur J Surg Oncol 2024;50:108307. Doi:10.1016/j.ejso.2024.108307.

Baseline MRF assessment

21. MRF invasion (incl. invasion by tumour bearing structures other than the primary tumour)

Draft statements 25, 26 and 27:

A margin of ≤ 1 mm between the MRF and the primary tumour, EMVI, or irregular nodules (representing tumour deposits or lymph nodes with extracapsular extension) is recommended as a criterion to diagnose an involved MRF

(evidence level III)

Reporting of a 'threatened' MRF (distance 1-2 mm) is not recommended

(evidence level III)

The MRF should be considered as non-involved in case of a ≤ 1 mm margin from lymph nodes (nonenlarged or enlarged) with smooth margins

(evidence level III)

Summary of relevant literature:

Al-Sukhni et al. (meta-analysis 2012; n=10 studies incl 986 patients with MRF assessment) reported a good accuracy to assess MRF status (sensitivity 77%; specificity 94%). The MERCURY group established that the 1 mm cut-off was the most predictive for local recurrence and that a larger distance to the MRF did not appear to identify patients at higher risk of local recurrence.

When looking at MRF-involvement by other structures than the primary tumour, Nagtegaal et al. (2002) showed that local recurrence rates in patients with a positive margin due to a positive lymph node were similar to local recurrence rates of patients with negative margins. In addition Shihab et al (2010) showed that mr-detected lymph nodes close to the mesorectal fascia are rarely a cause of margin involvement after total mesorectal excision. Evidence on the prognostic importance of margin involvement by tumour deposits or extramural vascular invasion (EMVI) is limited. Birbeck et al (2002; n=608) showed that margin involvement caused by EMVI or tumour deposits adds a 20% and 31% risk for local recurrence, respectively, versus a 42% risk for direct tumour invasion. In 2022 a multidisciplinary expert panel including radiologists, surgeons, pathologists and radiation oncologists (Lambregts 2022) considered these findings and agreed that the MRF should be considered involved in case of a ≤ 1 mm margin from either the primary tumour, EMVI, and irregular nodules (i.e., tumour deposits or lymph nodes with extracapsular extension) and as non-involved in case of lymph nodes (with smooth margins) close to the MRF

In summary, the panel has previously (in voting round 1) agreed on ≤ as the cut-off for MRF involvement. Current evidence suggests that the MRF should be considered involved in case of a ≤ 1 mm margin from the primary tumour, as well as EMVI and irregular nodules (i.e., tumour deposits or lymph nodes with extracapsular extension). It should be considered as non-involved in case of lymph nodes (with smooth margins) close to the MRF.

- Al-Sukhni et al. Diagnostic accuracy of MRI for assessment of T category, Lymph node metastases and circumferential resection margin involvement in patients with rectal cancer: a systematic review and meta-analysis.. Ann Surg Oncol 2012; 19: 2212-2223.
- Nagtegaal et al. Circumferential margin involvement is still an important predictor of local recurrence in rectal carcinoma: not one millimeter but two millimeters is the limit. Am J Surg Pathol 2002; 26(3):350-7
- Shihab et al. Magnetic resonance imaging-detected lymph nodes close to the mesorectal fascia are rarely a cause of margin involvement after total mesorectal excision. Br J Surg 2010; 97(9):1431–1436
- Birbeck et al. Rates of circumferential resection margin involvement vary between surgeons and predict outcomes in rectal cancer surgery. Ann Surg 2002; 235(4):449–457
- Lambregts et al. Current controversies in TNM for the radiological staging of rectal cancer and how to deal with them: results of a global online survey and multidisciplinary expert consensus. Eur Radiol 2022;32(7):4991-5003.
- Zhang et al. Diagnostic accuracy of MRI for assessment of T category and circumferential resection margin involvement in patients with rectal cancer: a meta-analysis. Dis Colon Rectum 2016;59:189-99.
- Taylor et al. One millimetre is the safe cut-off for magnetic resonance imaging prediction of surgical margin status in rectal cancer. Br J Surg 2011;98:872-879.

IV – Restaging (T2W)

Response assessment and tumour (yT) restaging

22. T2W MRI for yT-restaging after CRT

Draft statements 28, 29, 30 and 31:

T2-weighted MRI can accurately select patients with gross residual tumour who will require radical surgery (TME)

(evidence level I)

In patients with suspected residual tumour after neoadjuvant treatment, the yT-stage should be reported as an estimation (representing the maximum yT-stage) and encompass any fibrotically changed areas of the former tumour bed

(evidence level V)

The findings of T2-weighted MRI should be combined with those of DWI and endoscopy to assess a
(near) complete response after neoadjuvant treatment

(evidence level II)

In case of a suspected (near)complete response after neoadjuvant treatment, detailed yT-stage reporting is not recommended as it is unreliable and has no clinical implications

(evidence level III)

Summary of relevant literature:

Wei et al. (meta-analysis 2020; n=1262 patients) showed a sensitivity of 81% and specificity of 67% to detect yT3-4 tumours. The good results of MRI to identify patients with gross residual tumour (i.e., patients who will require radical resection) was also shown in a study by Haak et al (2020).

In a meta-analysis by Memon et al. (2015) 15 studies were included that evaluated yT-staging; overall accuracy ranged from 34-82% (average 52%). Overstaging was a frequent error in ypT0-1 tumours. ypT4 tumours were understaged rather than overstaged. Van der Paardt et al. (metaanalysis 2013) showed that overall sensitivity for yT-staging after CRT was 50% and specificity 91%. When looking at subgroups, the identification of ypT0 resulted in a sensitivity of 19% and specificity of 95%; distinguishing ypT0-2 from ypT3-4 led to a sensititivity of 55% and specificity of 90%.

El-Khababi et al. (2023; n=90) evaluated accuracy for MRI yT-staging after CRT in a multireader setting. yT-stage accuracy was highly dependent on the degree of fibrotic transformation of the tumour bed and concordance between the ymrT-stage and final ypT-stage at pathology was significantly higher in patients with predominant tumour compared to patients with predominant fibrosis (58% for patients with Mandard TRG 4-5 versus 41% for TRG 1-3). Overstaging was the main cause of error and occurred more frequently in the group with mainly fibrosis (38-44% versus 1355% in patients with predominant tumour).

Maas et al. (2015; n=50) showed that by combining T2-weighted MRI, diffusion and endoscopy the highest performance can be achieved to identify complete responders. Adding DWI and endoscopy increases the sensitivity to detect a CR. This combined method (MRI + endoscopy) has been adopted as the recommended method of response evaluation by clinical guidelines and was shown to represent the routine method of response evaluation in the vast majority of centers offering watchand-wait within the international watch-and-wait database (IWWD; van der Valk et al. 2018).

In summary, T2W MRI has moderate accuracy for yT-restaging with particularly poor sensitivity to identify complete responders. yT-stage accuracy is negatively correlated with the degree of fibrosis. Overstaging is the main source of error. Considering these limitations, yT-stage should be reported as an estimation. In patients with a (near)complete response who are potential candidates for organ-preservation it may be avoided all together as it will have no clinical implications. In patients scheduled to undergo surgery, yT-stage estimation should encompass all areas potentially at risk to harbour tumour (incl. fibrosis) as these will need to be resected at surgery. To diagnose a complete response, MRI should be combined with endoscopy.

- Wei et al. The Diagnostic Accuracy of Magnetic Resonance Imaging in Restaging of Rectal Cancer After Preoperative Chemoradiotherapy: A Meta-Analysis and SystematicReview. J Comput Assist Tomogr 2020;44:102-110.
- Haak et al. Selection of Patients for Organ Preservation After Chemoradiotherapy: MRI Identifies Poor Responders Who Can Go Straight to Surgery. Ann Surg Oncol 2020;27:2732-2739.
- Memon et al. Systematic review and meta-analysis of the accuracy of MRI and ERUS in the restaging and response assessment of rectal cancer following neoadjuvant therapy. Colorect Dis 2015;17:748761.
- El-Khababi et al. Sense and non-sense of yT-staging on MRI after chemoradiotherapy in rectal cancer. Colorect Dis 2023;25:1878-1887.
- Maas et al. Assessment of Clinical Complete Response After Chemoradiation for Rectal Cancer with Digital Rectal Examination, Endoscopy, and MRI: Selection for Organ-Saving Treatment. Ann Surg Oncol 2015; 22:3873-80. doi: 10.1245/s10434-015-4687-9.

- Van der Valk et al. Long-term outcomes of clinical complete responders after neoadjuvant treatment for rectal cancer in the International Watch & Wait Database (IWWD): an international multicentre registry study. Lancet 2018;391:2537-2545.

23. mrTRG to assess a complete response

Draft statements 32:

mrTRG is useful to estimate the overall degree of response (good vs poor) but is not accurate to identify patients with a complete response

(evidence level II)

Summary of relevant literature:

mrTRG is an adaptation of the pathologic tumour regression grading system proposed by Mandard et al. It quantifies fibrosis relative to residual tumour. A meta-analysis by Jang et al. (2020; n=916 patients) showed that sensitivity of mrTRG1 to identify CR was 32%, with a specificity of 94%.
mrTRG1-2 had a sensitivity of 70% and specificity of 62% to identify CR. The authors concluded that mrTRG is of limited clinical value to select patients for organ-preserving treatments after CRT. This was confirmed in a more recent multireader study El-Khababi et al. (2023; n=90) who showed that mrTRG has a sensitivity of 57% and specificity of 64% to identify a CR.

A meta-analysis by Siddiqui et al. (2016) showed that mrTRG has value to predict long-term prognosis and that mrTRG1-3 predicts good long-term outcome and mrTRG4-5 poor long-term outcome.

In summary, mrTRG has been demonstrated to have prognostic value and appears useful to make a general estimation of the degree of response. As a stand along score it is, however, not reliable to diagnose a complete response.

- Jang et al. MR tumour regression grade for pathological complete response in rectal cancer post neoadjuvant chemoradiotherapy: a systematic review and meta-analysis for accuracy. Eur Radiol 2020; 30(4): 2312-2323.
- El-Khababi et al. Comparison of MRI response evaluation methods in rectal cancer: a multicentre and multireader validation study. Eur Radiol 2023;33(6):4367-4377
- Siddiqui et al. Defining response to radiotherapy in rectal cancer using magnetic resonance imaging and histopathological scales World J Gastroenterol. 2016 Oct 7;22(37):8414-8434. doi: 10.3748/wjg.v22.i37.8414.

24. Other response grading systems (modified TRG, split scar, DWI patterns)

Draft statement 33:

Response evaluation after neoadjuvant CRT should include an estimation of the degree and pattern of fibrosis combined with the presence and pattern of diffusion restriction

(evidence level III)

Summary of relevant literature:

The split scar sign is a morphologic sign describing a specific layered appearance of the fibrotic scar on T2W MRI. A recent meta-analysis by Torrit et al (2024; n=377) showed that the split scar sign has a high pooled specificity (92%) but low sensitivity (62%) to detect complete responders.

The modified TRG method (Lee 2017; n=118) combines the degree of fibrosis on T2W MRI with the presence of diffusion restriction in a 3-point score (complete regression/intermediate regression/poor regression). The authors showed an overall accuracy of 73% which was significantly higher than for mrTRG (38%). A comparable approach was adopted by Haak et al. (2020; n=62) who showed that using this approach poor responders who will definitely require radical resection can be accurately selected by radiologists of varying expertise. Lambregts et al. (2018; n=222) combined morphologic patterns of fibrosis with specific signal patterns of DWI and showed an overall accuracy of 88%.

El-Khababi et al. (2023; n=90) compared the mrTRG, split scar sign, modified mrTRG, and diffusion pattern approach by Lambregts to assess response after CRT in a single study involving 22 radiologists. They found that sensitivity to diagnose a CR was generally low (36-40%). Specificity ranged between 64-82%. Reader experience and image quality both had a significant effect on performance. IOA was lower for the split scar sign (0.18 versus 0.39-43 for the other methods). Methods incorporating DWI were preferred by the majority of readers and showed the most favourable results when combining performance, IOA and reader preference. The combined assessment of fibrosis (on T2W MRI) and DWI has also been adopted by previous ESGAR guidelines, and other radiological guidelines (e.g., SAR, GRECCAR)

In summary, literature supports the use of grading systems that incorporate patterns of fibrosis on T2W MRI with findings of DWI, in specific to help diagnose patients with a complete response after neoadjuvant treatment.

- Torri et al. Split scar sign to predict complete response in rectal cancer after neoadjuvant chemoradiotherapy: systematic review and meta-analysis. Eur Radiol 2024;34:3874-3881.
- Lee et al. Modified 3-Point MRI-Based Tumour Regression Grade Incorporating DWI for Locally Advanced Rectal Cancer. AJR 2017;209:1247-1255.
- Lambregts et al. A Pattern-Based Approach Combining Tumour Morphology on MRI With Distinct Signal Patterns on Diffusion-Weighted Imaging to Assess Response of Rectal Tumours After Chemoradiotherapy. Dis Colon Rectum;2018:328-337.
- Haak et al. Selection of Patients for Organ Preservation After Chemoradiotherapy: MRI Identifies Poor Responders Who Can Go Straight to Surgery. Ann Surg Oncol 2020;27:2732-2739.
- El-Khababi et al. Comparison of MRI response evaluation methods in rectal cancer: a multicentre and multireader validation study. Eur Radiol 2023;33(6):4367-4377

Nodal (yN) restaging

25. T2W MRI for yN-staging

Draft statements 34 and 35:

A size cut-off of 5 mm (short axis) may be used to restage mesorectal lymph nodes following neoadjuvant treatment, while being mindful of its limitations

(evidence level III)

When considering patients for organ preservation, MRI may be used to monitor nodal growth (‘test of time’) and identify yN+ disease

(evidence level V)

Summary of relevant literature:

A meta-analysis by Al-Sukhni et al. (2012) evaluated the performance of MRI for yN-staging and reported a sensitivity of 77% and specificity of 71%. It should be noted that nodal criteria differed between included studies (and were unspecified for some); the majority used a 5 mm cut-off (shortaxis diameter), with or without morphologic criteria. Memon et al (2015) showed in another metaanalysis of 15 studies that nodal restaging criteria varied widely. Overall accuracy was 72%, with over- and understaging occurring in 16%. Van der Paardt et al. showed a sensitivity of 77% and specificity of 60% for ycN-staging, with a higher performance when a lesion-per-lesion based analyses was performed (sensitivity of 91% and specificity of 73%). The 5 mm cut-off yielded a sensitivity of 72% and specificity of 71%.

As shown by Haak et al (2021; n=1898), the risk for residual ypN+ disease is correlated with the ypTstage and is low (7%) in patients with a complete response of their primary tumour (ypT0). Furthermore, results from the international watch-and-wait database (van der Valk et al 2018; n=1009) show that the incidence of nodal regrowths during watchful waiting is very low (3%). These findings seem to warrant a more hesitant approach to determine treatment decisions (i.e. organ preservation) based on yN-stage in patients that show a good response of their primary tumour. Experts suggest that the ‘test of time’ can be used as an additional diagnostic tool to monitor nodes after neoadjuvant therapy / during watch-and-wait.

In summary, MRI has a moderate-good performance to restage nodes after neoadjuvant treatment. The 5 mm cutoff proposed in the previous edition of the ESGAR guidelines may still be used in practice, while acknowledging its limitations. In patients with a good response of their primary tumour, who may be candidates for organ-preservation, equivocal lymph nodes may be monitored
(‘test of time’).

- Al-Sukhni et al. Diagnostic accuracy of MRI for assessment of T category, Lymph node metastases and circumferential resection margin involvement in patients with rectal cancer: a systematic review and meta-analysis.. Ann Surg Oncol 2012; 19: 2212-2223.
- Memon et al. Systematic review and meta-analysis of the accuracy of MRI and ERUS in the restaging and response assessment of rectal cancer following neoadjuvant therapy. Colorect Dis 2015;17:748761.
- Van der Paardt et al. Patients who undergo preoperative chemoradiotherapy for locally advanced rectal cancer restaged by using diagnostic MR imaging: a systematic review and meta-analysis. Radiology 2013; 269: 101–12
- Haak et al. Prevalence of nodal involvement in rectal cancer after chemoradiotherapy. Br J Surg 2021;108:1251-1258.
- Van der Valk et al. Long-term outcomes of clinical complete responders after neoadjuvant treatment for rectal cancer in the International Watch & Wait Database (IWWD): an international multicentre registry study. Lancet 2018;391:2537-2545.

26. Lateral nodal restaging after CRT

Draft statement 36:

There are currently no recommended size thresholds or other criteria for lateral nodal restaging after neoadjuvant treatment

(level of evidence V)

Summary or relevant literature:

In 2019 the lateral node study consortium (Ogura et al; n=741) showed that shrinkage of initially enlarged (≥7 mm) nodes to ≤ 4 mm after CRT represent a safe margin to avoid lateral lymphadenectomy after neoadjuvant treatment. Risk for lateral nodal recurrence for nodes > 4 mm on restaging MRI was high (>50%) especially for internal iliac nodes; for obturator nodes a size threshold of > 6 mm was proposed. Limitations of this study included the underpowered sample and heterogeneous preoperative treatment regimens (i.e. radiotherapy with and without chemotherapy, different doses, different intervals from treatment to restaging and final surgery). Based on these limitations, recommendations from a multidisciplinary consensus panel from 2022 (Lambregts et al.) stated that the size criteria proposed by Ogura et al. should not (yet) be adopted as the evidence provided is too preliminary. The panel also acknowledged that at the time of publication no alternative criteria were available.

More recently (2024; n=284) van Geffen et al. published a national (Dutch) study including patients who underwent neoadjuvant treatment and had visible lateral LNs (> 5 mm) on baseline imaging. Results showed that in patients with intermediate sized nodes (5-7 mm) and malignant features at baseline, disappearance of these malignant features on restaging MRI was prognostically more favourable compared to patients in whom malignant features persisted on restaging MRI (lateral nodal recurrence risk 7% versus 13%), though patient numbers in these subgroups were low (n=1719) and no firm conclusions could be drawn. In patients with enlarged lymph nodes (≥ 7 mm) the risk for lateral nodal recurrence increased with the number of enlarged nodes encountered at baseline and with the presence of multiple malignant features; however, no data was provided on the disappearance of these malignant features after neoadjuvant treatment in this subgroup. Moreover, the study by van Geffen provides no recommendations on size-thresholds after neoadjuvant treatment. In a paper by Sluckin et al (from the same Dutch group) it was shown that lateral nodal downsizing after neoadjuvant treatment had no impact on lateral local recurrence rates, suggesting that size after (C)RT should not be taken into account.

<u>In summary</u>, published data from the lateral node study consortium and Dutch snapshot research group contradict each other and are limited by low number of events (i.e. low number of patients with lateral nodal recurrences) and heterogeneous neoadjuvant treatment regimens of included study patients. As such, there are to date no criteria to restage lateral lymph nodes after CRT supported by a sufficient body of evidence.

- Ogura et al. Lateral Nodal Features on Restaging Magnetic Resonance Imaging Associated With Lateral Local Recurrence in Low Rectal Cancer After Neoadjuvant Chemoradiotherapy or Radiotherapy. JAMA Surg. 2019 Sep 1;154(9):e192172. doi: 10.1001/jamasurg.2019.2172.
- Lambregts et al. Current controversies in TNM for the radiological staging of rectal cancer and how to deal with them: results of a global online survey and multidisciplinary expert consensus. Eur Radiol 2022;32(7):4991-5003.

- Van Geffen et al. Value of Size and Malignant Features of Lateral Lymph Nodes in Risk Stratification at Lateral Local Recurrence of Rectal Cancer: A National Cohort Study. J Natl Compr Canc Netw. 2024 Feb;22(1):17-25. doi: 10.6004/jnccn.2023.7081.
- Sluckin et al. Prognostic Implications of Lateral Lymph Nodes in Rectal Cancer: A Population-Based Cross-sectional Study With Standardized Radiological Evaluation After Dedicated Training. Dis Colon Rectum 2024; 67: 42–53

yEMVI assessment

27. Performance of T2W MRI for yEMVI assessment

28. Criteria to stage tumours as yEMVI+ after neoadjuvant treatment

Draft statement 37

The same grading system should by applied for both primary EMVI staging and yEMVI staging following neoadjuvant treatment

(evidence level II)

Summary of relevant literature:

Chand et al. (2014; n=62) introduced a post-CRT EMVI grading system (mr-v-TRG) that is more or less in line with the mrTRG and quantifies the amount of fibrosis vs tumour signal compared to pretreatment imaging on a 5-point scale. Patients were categorized to mr-v-TRG1-3 (good responder) vs mr-v-TRG4-5 (poor responder). mr-v-TRG4-5 had a worse prognosis. This system has not been validated or widely adopted by other groups.

Another group (Chand et al. 2015; n=188) looked into post-CRT staging of mr-based EMVI (ymrEMVI) In 188 patients MRI detected more patients with persistent EMVI than histopathology (53 vs 19%), indicating overstaging of the presence of EMVI. In long-term outcome analyses both mr-detected and histopathological yEMVI+ were predictive for poor prognosis. The findings were independent of yT and yN-stage.

Meta-analyses (Chen 2021; Tan 2021) that looked at the prognostic value of ymrEMVI confirmed the worse prognosis in ymrEVMI+ patients: DFS HR 1.93-2.24 A meta-analysis by Rouleau-Fournier (2022) furthermore showed that persistent ymrEMVI is also associated with a higher risk for R1 resection (HR 2.95).

In summary, literature has shown the prognostic significance of persistent yEMVI after neoadjuvant treatment. Apart from one small study (Chand 2014) no specified criteria have been reported for yEMVI grading after CRT and the most commonly adopted approach is to use the same criteria also used for primary EMVI staging.

- Chand et al. Extramural venous invasion is a potential imaging predictive biomarker of neoadjuvant treatment in rectal cancer. Br J Cancer 2014; 110:19-25
- Chand et al. The Prognostic Significance of Postchemoradiotherapy High-resolution MRI and Histopathology Detected Extramural Venous Invasion in Rectal Cancer. Ann Surg 2015;261:473-479
- Chen et al. The prognostic value of MRI-detected extramural vascular invasion (mrEMVI) for rectal cancer patients treated with neoadjuvant therapy: a meta-analysis. Eur Radiol 2021;31:8827-8837.

- Tan et al. Prognostic Importance of MRI-Detected Extramural Venous Invasion in Rectal Cancer: A Literature Review and Systematic Meta-Analysis. Int J radiat Oncol Biol Phys 2021;111:385-394.
- Rouleau Fournier, et al. Oncologic Outcomes Associated With MRI-detected Extramural Venous Invasion (mrEMVI) in Rectal Cancer: A Systematic Review and Meta-analysis. Ann Surg 2022;275:303314.

yMRF assessment

29. Performance of T2W MRI for yMRF assessment

Draft statements

N/A

Summary of relevant literature:

A meta-analysis by Memon et al. included 5 restaging studies on MRI for yMRF status and found a high NPV of 98%, but a high rate of overstaging (PPV 45%).

- Memon et al. Systematic review and meta-analysis of the accuracy of MRI and ERUS in the restaging and response assessment of rectal cancer following neoadjuvant therapy. Colorect Dis 2015;17:748761.

T2W Other

30. Criteria for restaging after non-standard neoadjuvant treatment

Draft statement 38:

The same criteria to assess response after neoadjuvant (chemo)radiotherapy are also recommended for patients undergoing alternative neoadjuvant treatments (e.g., TNT)

(evidence level V)

Summary of relevant literature:
The literature search retrieved no studies investigating specific methods for response evaluation related to alternative neoadjuvant treatments other than standard of care CRT. Total neoadjuvant treatment (TNT) is currently the most commonly used alternative treatment for high risk tumours.

Hall (2023; n=121) suggested a limited performance for MRI to predict a complete response after TNT (PPV 40%).

In summary, there is to date no evidence supporting alternative MRI grading or assessment methods to assess response in patients undergoing TNT (or other alternative neoadjuvant treatments). As such from a practical point of view, the same criteria to assess response after standard of care neoadjuvant (chemo)radiotherapy are also recommended for patients undergoing alternative neoadjuvant treatments.

- Hall WA, et al. Prospective Correlation of Magnetic Resonance Tumour Regression Grade With Pathologic Outcomes in Total Neoadjuvant Therapy for Rectal Adenocarcinoma. J Clin Oncol. 2023 Oct 10;41(29):4643-4651. doi: 10.1200/JCO.22.02525. Epub 2023 Jul 21.

31. - Criteria to assess response in mucinous tumours - Prognostic significance of mucinous transformation in primarily non-mucinous tumours

Draft statements 39 and 40:

It is recommended to use the same response criteria after neoadjuvant treatment for both solid tumours and mucinous tumours

(evidence level IV

Mucinous degeneration in primarily non-mucinous response should not be regarded as a sign of non-response.

(evidence level II)

Summary of relevant literature:
A meta-analysis from Reynolds et al. (2020; 11 studies, n=1947 patients) showed that mucinous degeneration (in primarily non-mucinous tumours) is not associated with pathologic degree of tumour response, recurrence or survival. These results were confirmed by a more recent singlecenter study (Miranda 2023; n=201). A large study by Nagtegaal (2004; n=1304) showed that patients who develop mucinous components post-RT ("induced mucinous carcinoma") have a significantly better prognosis compared to primarily mucinous tumours (2 year recurrence free interval 92 versus 39%).

Only 1 small study attempted to develop a specific grading system to assess response in mucinous tumours (Park 2017; n=59). This modified TRG (TRG 1, no identifiable residual lesion; TRG 2, no residual soft tissue, only pure mucin and/or fibrosis; TRG 3, good response of soft tissue; TRG 4, all tumours that do not meet criteria do TRG 1–3; and TRG 5 no response or progression) showed significant correlations with pathologic TRG but IOA was moderate (kappa 0.40) and results have not been validated or tested in terms of diagnostic performance.

In summary, primarily mucinous tumour typically show a worse response to neoadjuvant treatment compared to non-mucinous tumours and MRI has a poor performance to distinguish between acellular mucin and mucin still containing viable tumour after treatment. There is no compelling evidence to support alternative response criteria or grading systems for mucinous tumours. Mucinous degeneration (synonyms: colloid response, mucin pool formation) in primarily nonmucinous tumours is not associated with tumour response, recurrence or survival

- Reynolds IS, et al. Mucin Pools Following Neoadjuvant Chemoradiotherapy for Rectal Cancer: A Marker of Response or Epiphenomenon? Am J Surg Pathol. 2020 Feb;44(2):280-287. doi: 10.1097/PAS.0000000000001373.
- Miranda J, et al. Mucinous Degeneration on MRI After Neoadjuvant Therapy in Patients With Rectal Adenocarcinoma: Frequency and Association With Clinical Outcomes. AJR Am J Roentgenol 2023 Aug;221(2):206-216. doi: 10.2214/AJR.23.29002

- Nagtegaal I, et al. Morphological changes in tumour type after radiotherapy are accompanied by changes in gene expression profile but not in clinical behavior. J Pathol 2004 Oct;204(2):183-92. doi: 10.1002/path.1621.
- Park SH, et al. Rectal Mucinous Adenocarcinoma: MR Imaging Assessment of Response to Concurrent Chemotherapy and Radiation Therapy—A Hypothesis-generating Study. Radiology 2017 Oct;285(1):124-133. doi: 10.1148/radiol.2017162657.

---

32. How to incorporate fibrosis when assessing tumour height and length after CRT

Draft statement 41:

Tumour height and length measurements after neoadjuvant treatment should encompass the fibrotic remnants of the tumour bed

(evidence level V)

Summary of relevant literature:

There are no studies that have specifically addressed the inclusion (or exclusion) of fibrotic remnants of the tumour bed in height and length measurements after neoadjuvant treatment, or its impact on surgical planning, surgical outcomes and long-term prognosis. The French GRECCAR/GRERCAR consensus guidelines on imaging for restaging of rectal cancer propose in their reporting template to include fibrosis in the measurements, as – in case of surgical resection – the fibrotic remnants should be included in the resection.

- Nougaret S, et al. MRI restaging of rectal cancer: The RAC (Response-Anal canal-CRM) analysis joint consensus guidelines of the GRERCAR and GRECCAR groups. Diagn Interv Imaging. 2023 JulAug;104(7-8):311-322. doi: 10.1016/j.diii.2023.02.002.

---

33. Lexicon to classify tumours into different response groups after CRT

Draft statement 42:

A restaging MRI report should include an overall classification of response to help inform

2. Small residual tumour (i.e., patients potentially eligible for local treatment)
3. Gross residual tumour (i.e., patients requiring radical resection)

(evidence level V)

further treatment planning:

1. (Near) CR (i.e., patients potentially eligible for watchful waiting)

Summary of relevant literature:

Terminology used to classify response groups after neoadjuvant treatment varies widely in published literature, also depending on whether based on imaging and/or endoscopic evaluation. Four main groups (based on imaging and endoscopy) have been reported defined based on the different treatment implications :

- Complete response (CR): eligible for organ preservation with watchful waiting
- Near CR: possible or almost complete response that may evolve into a CR (and become eligible for watchful waiting) with a longer waiting interval
- Small residual tumour: potentially eligible for organ preservation by means of additional local treatment (e.g., local excision, local boost RTx)
- Gross residual tumour: requiring radical surgery (TME or beyond)

Currently, no uniform criteria exist to define a near CR versus small residual tumour, but a consensus-based process by Custers et al. (2024) did recommend some helpful features. The consensus panel from this study furthermore agreed a near CR label should be temporary (i.e. used only at the first response assessment(s)) and no longer be used after 6 months following neoadjuvant CRT. It should be noted that the criteria stipulated in that consensus paper are not only radiology based, but rely heavily on endoscopy findings.

The SAR Rectal cancer lexicon proposes 2 categories: (near) CR or incomplete response.

In summary, there is a lack of evidence on which lexicon to use to stratify patients into different response groups. However, experts agree that classifying patients into different response groups is important to guide treatment planning, in particular to discern patients who are potentially eligible candidates for organ preservation (either through watchful waiting or local additional therapy) from patients that need radical surgery. From the perspective of treatment implications we propose a 3way classification where we have grouped complete and near-complete (=potential complete responders) together considering these may both proceed to be eligible candidates for watchful waiting:

1. (Near) CR, i.e., patients potentially eligible for watchful waiting
2. Small residual tumour, i.e., patients potentially eligible for local treatment
3. Gross residual tumour, i.e., patients requiring radical resection (TME or beyond TME)

- Custers et al. An International Expert-Based Consensus on the Definition of a Clinical Near-Complete Response After Neoadjuvant (Chemo)radiotherapy for Rectal Cancer. Dis Colon Rectum 2024;67:782-795.
- Lee et al. Rectal cancer lexicon 2023 revised and updated consensus statement from the Society of Abdominal Radiology Colorectal and Anal Cancer Disease-Focused Panel. Abdom Radiol 2023;48:2792-2806.
- Ou et al. Sense and non-sense of imaging in the era of organ preservation for rectal cancer. Br J Radiol 2023; 96:20230318. doi: 10.1259/bjr.20230318

V – Diffusion-weighted imaging

Baseline staging

34. -39. Performance of DWI for T-, N-, EMVI and MRF and to discern LNs from tumour deposits?

Draft statements 43 and 44:

DWI is not recommended for baseline T-staging, assessment of EMVI or MRF involvement

(evidence level II-V)

Use of DWI is optional to detect and localize lymph nodes, but DWI is not recommended for characterization of lymph nodes or to differentiate between lymph nodes and tumour deposits

(evidence level III)

Summary of relevant literature:
Two small retrospective studies (Lu 2016, n=60; Feng 2014, n=46) assessed the value of DWI in addition to T2W MRI (compared to T2W only) for T-staging and found no significant benefit in terms of diagnostic accuracy.

Two studies (Fornell-Perez 2020, n=54; Ahn 2019, n=79) retrospectively assessed the value of DWI
(compared to T2W MRI) to assess EMVI. Ahn et al. found no statistically significant effect. FornellPerez et al. only found significant results for expert radiologists, with higher specificity for T2W+DWI compared to T2W only (87 versus 76%) but at the cost of a lower sensitivity (48 versus 57%) and with similar overall accuracy (0.67 for T2W as well as for T2W+DWI). No significant effects were found for less experienced radiologists and residents.

No studies were found reporting on DWI for assessment of MRF

As outlined in a review by Schurink et al. (2019), DWI enhances the detection of lymph nodes, but reported PPVs for high DWI signal to predict N+ nodes are low (±50%). Majority of nodal DWI studies focused on quantitatively measuring nodal ADC values. Sensitivities to characterize nodes using ADC (based on retrospectively determined threshold values) ranged from 67-88% and specificities from 60-97%. Big variations and considerable overlap between malignant and benign nodes were found between studies. In a meta-analysis from 2021 Surov et al. reported that no reliable ADC thresholds could be derived from available literature to discern metastatic from non-metastatic lymph nodes.

One small study (Xu et al 2023; n=30 with node-by-node rad-pat matching) compared morphologic, ADC and DCE features between lymph nodes and tumour deposits. ADC max/min/mean values were not statistically different between TDs and metastatic lymph nodes. Only the lesion-to-primary tumour ratio showed significant differences between metastatic lymph nodes and TDs for ADC min and mean. No further evidence exists on the value of DWI to differentiate between lymph nodes and tumour deposits.

In summary, there is limited evidence supporting a role for DWI for baseline T-, N-, EMV and MRF staging, nor to discern lymph nodes from tumour deposits.

- Lu et al. Preoperative diffusion-weighted imaging value of rectal cancer: preoperative T staging and correlations with histological T stage. Clin Imaging 2016:40:563-568
- Feng et al. T staging of rectal cancer: Accuracy of diffusion-weighted imaging compared with T2weighted imaging on 3.0 tesla MRI. Journal of digestive diseases 2014;15:188-194
- Fornell-Perez et al. Primary and post-chemoradiotherapy MRI detection of extramural venous invasion in rectal cancer: the role of difusion-weighted imaging. La radiologica medica 2020;125:522530
- Ahn et al. Added value of diffusion-weighted imaging for evaluation of extramural venous invasion in patients with primary rectal cancer. Br J Radiol 2019; 92: 20180821.

- Schurink et al. Diffusion-weighted imaging in rectal cancer: current applications and future perspectives. Br J Radiol 2019;92:20180655
- Surov et al. Apparent diffusion coefficient cannot discriminate metastatic and non-metastatic lymph nodes in rectal cancer: a meta-analysis. Int J Colorect Dis 2021;36:2189-2197
- Xu et al. Distinguishing mesorectal tumour deposits from metastatic lymph nodes by using diffusionweighted and dynamic contrast-enhanced magnetic resonance imaging in rectal cancer. European Radiology (2023) 33:4127–4137

Restaging

40. Performance of DWI (visual assessment) to assess a complete response vs residual tumour after neoadjuvant treatment

Draft statements 45 and 46

DWI is mandatory for tumour response assessment as it significantly enhances the performance of MRI to discern between residual tumour and complete response after neoadjuvant treatment.

(evidence level I-II)

DWI should be assessed in conjunction with T2W MRI

(evidence level I-II)

Summary of relevant literature:
As outlined in a systematic review by Schurink et al (including papers up to 2016), visual assessment of DWI significantly improves the performance of MRI to discern yT0 versus yT+ disease as shown by 7 different publications with patient numbers ranging from n=19-120 with reported AUCs 0.70-0.96 for T2W MRI + DWI versus 0.67-0.85 for T2W MRI only (statistically significant effect in majority of reports). In a study including 251 patients published in 2021 (following the review by Schurink) Chandramohan et al. reported similar results with an AUC of 0.53 for T2W MRI versus AUC 0.88 for T2W + DWI.

Lee (2017) and Haak (2020) reported similar modifications of the mrTRG incorporating the presence/absence of high DWI signal. In a multireader study including 22 radiologists using a multicenter MRI dataset (el Khababi et al 2023; n=90) this modified TRG resulted in a sensitivity of 79%, specificity of 40% and overall accuracy of 67% to discern patients with residual tumour from complete responders. Lambregts et al. (2018; n=222) combined DWI with specific morphologic patterns and reported a sensitivity of 94%, specificity 77% and overall accuracy of 88%. In the multireader validation study by El Khababi et al. this approach resulted in a sensitivity of 82%, specificity 37% and accuracy of 68%. These results indicate that the main risk of DWI is overstaging (i.e. false positive DWI signal resulting in misclassification of complete responders as having residual tumour). The study el Khababi furthermore showed that image quality and experience level of readers have a significant impact on diagnostic performance.

In summary, it has convincingly been shown that DWI improves the diagnostic performance of MRI to differentiate between a complete response and residual tumour in the restaging

setting. DWI should be assessed in conjunction with T2W MRI. The main risk of DWI is false positive results (i.e. misclassifying complete responders as having residual tumour).

- Schurink et al. Diffusion-weighted imaging in rectal cancer: current applications and future perspectives. Br J Radiol 2019;92:20180655

- Chandramohan et al. Diffusion weighted imaging improves diagnostic ability of MRI for determining complete response to neoadjuvant therapy in locally advanced rectal cancer. European Journal of Radiology Open 7 (2020) 100223

- Lee et al. Modified 3-point MRI-based tumour regressuion grade incorporating DWI for locally advanced rectal cancer. AJR Am J Roentgenol 2017;209:1247-1255
- Haak et al. Selection of Patients for Organ Preservation After Chemoradiotherapy: MRI Identifies Poor Responders Who Can Go Straight to Surgery. Ann Surg Oncol 2020;27:22732-2739
- El Khababi et al. Comparison of MRI response evaluation methods in rectal cancer: a multicenter and multireader validation study. Eur Raiol 2023;33:4367-4377

---

41. -44. Performance of DWI for yT-, yN-, yEMVI, yMRF staging

Draft statements 47, 48 and 49:

DWI is not recommended for yT-staging, except to assess yT0 versus yT+

(evidence level V)

DWI is not recommended or yN-staging after neoadjuvant treatment

(evidence level III)

DWI may be of added value to assess yEMVI and yMRF status after CRT

(evidence level III)

Summary of relevant literature:
In a meta-analysis by van der Paardt et al (2013), pooled sensitivity for predicting response (defined as either ypT0, ypT0-2 or T-downstaging compared to primary staging) was significantly higher for studies that included DWI in the MR protocol compared to studies that did not (83.6% *vs* 50.4%). A second meta-analysis by Wu et al (2013) also showed an improvement (albeit non-significant) to assess response for studies including DWI compared to studies focusing on T2W MRI, but this metaanalysis grouped studies focusing on visual DWI as well as ADC analysis, and included studies with different response outcomes (complete response, response not otherwise specified), making it difficult to draw more specific conclusions on the benefit of DWI. No studies have specifically focused on the use of DWI for more detailed yT-restaging.

Lambregts et al performed a node-by-node analysis (n=30; 157 nodes) and found that nodal signal intensity on DWI did not differ between yN- and yN+ nodes (AUC 0.52-0.64). Two studies assessed the use of DWI to predict lymph node eradication (*i.e.* yN0 stage) after CR. van Heeswijk (n=90) reported that the visual absence of nodes on DWI after CRT could

predict yN0 with a sensitivity of 100%, but very low specificity of only 14% (indicating many false positives). Ryu et al (n=95) used a confidence score to predict lymph node eradication after CRT with and without DWI and found no improvement in diagnostic performance for DWI compared to T2W-MRI with AUCs in the same range of 0.77–0.80. Kim et al. (2015) showed that nodal ADC measurements had no added benefit compared to nodal size to characterize lymph nodes after CRT. Surov et al. (meta-analysis from 2021 including 44% patients after CRT) reported that no reliable ADC thresholds could be derived from available literature to discern metastatic from non-metastatic lymph nodes.

Crimi et al. (2023; n=103) compared DWI, T2W-MRI and contrast-enhanced T1W sequences to discern yEMVI+ tumours after CRT and reported that AUC of DWI (0.73) was significantly higher than that of T2W-MRI (AUC 0.61) and ce-T1W (AUC 0.62). Fornell-Perez (2020; n=46) showed that expertradiologists showed significantly better results for DWI versus T2W-MRI to assess yEMVI+ (accuracy 0.85 versus 0.75), but no significant difference was found for general radiologists or residents.

A single report by Park et al. (n=45) evaluated the use of DWI in addition to *T*2W-MRI to predict tumour clearance of the MRF after neoadjuvant CRT. The authors reported a significantly improved performance after the addition of DWI (AUC 0.92–0.96) compared to use of only *T*2W MRI (AUC 0.77–0.85).

In summary, there is no evidence showing that DWI has any value for yT- of yN- restaging. DWI may have some benefit to assess yEMVI and yMRF after CRT, though evidence to support this is limited.

- Van der Paardt et al. Patients who undergo preoperative chemoradiotherapy for locally advanced rectal cancer restaged by using diagnostic MR imaging: a systematic review and meta-analysis. Radiology 2013; 269: 101–12
- Wu et al. Is there a benefit in using magnetic resonance imaging in the prediction of preoperative neoadjuvant therapy response in locally advanced rectal cancer? Int J Colorectal Dis 2013; 28: 1225– 38.
- Lambregts et al. Value of ADC measurements for nodal staging after chemoradiation in locally advanced rectal cancer—a per lesion validation study. Eur Radiol 2011; 21: 265–73
- van Heeswijk MM, et al. DWI for Assessment of Rectal Cancer Nodes After Chemoradiotherapy: Is the Absence of Nodes at DWI Proof of a Negative Nodal Status? American Journal of Roentgenology 2017; 208: W79–W84
- Kim et al. Apparent diffusion coefficient for lymph node characterization after chemoradiation therapy for locally advanced rectal cancer. Acta Radiol 2015;56:1446-1453
- Ryu et al. Diffusion-weighted imaging for evaluating lymph node eradication after neoadjuvant chemoradiation therapy in locally advanced rectal cancer. Acta radiol 2016; 57: 133–41
- Surov et al. Apparent diffusion coefficient cannot discriminate metastatic and non-metastatic lymph nodes in rectal cancer: a meta-analysis. Int J Colorect Dis 2021;36:2189-2197
- Crimi et al. Diagnostic accuracy of state-of-the-art rectal MRI sequences for the diagnosis of extramural vascular invasion in locally advanced rectal cancer after preoperative chemoradiotherapy: dos or maybes? European Radiology 2023; 33:6852–6860
- Fornell-Perez et al. Primary and post-chemoradiotherapy MRI detection of extramural venous invasion in rectal cancer: the role of difusion-weighted imaging. La radiologica medica 2020;125:522530
- Park MJ, et al. Locally Advanced Rectal Cancer: Added Value of Diffusion-weighted MR Imaging for Predicting Tumour Clearance of the Mesorectal Fascia after Neoadjuvant Chemotherapy and Radiation Therapy. Radiology 2011; 260: 771–80

VI – Miscellaneous

45. Sigmoid take-off (STO) to discern rectal from sigmoid cancers

Draft statements 50 and 51:

It is recommended to include the relation of the tumour to the sigmoid take-off (STO) in the MRI report to discern rectal from sigmoid cancers

(evidence level V)

It is recommended to classify tumours with a lower tumour border starting below the STO as rectal, and tumours starting above the STO as sigmoid.

(evidence level V)

Summary of relevant literature:
An international multidisciplinary expert panel agreed with 81% consensus on the STO as the preferred landmark to separate the rectum from the sigmoid (d'Souza 2019). Hazen et al (2021 and 2023) reported on implementation of the STO in the Netherlands after adoption of the STO in the Dutch guidelines; after one year ± half to one third of surveyed hospitals adopted the STO. Three reports (all from the Netherlands) assessed the potential impact of using the STO on treatment planning, compared to 'old' landmarks. Burghgraef (2023;n=1307) and Hazen (2024;n=2784) analyzed patients from the Dutch colorectal Cancer Audit that were originally diagnosed as having rectal cancer, and reported that when re-reviewed using the STO, 11-13% would be reclassified as sigmoid cancers. Approximately half of these patients (6-7% of the total cohort) would in retrospect have had received a different treatment according to current guidelines. A similar study (Bogveradze 2021; n=155) zoomed in on patients with tumours located near the rectosigmoid junction, and showed that in this subgroup use of the STO changed the classification from rectal to sigmoid in 28% of the cases, with potential implications for treatment planning in 23%.

Bogveradze furthermore showed that IOA to assess the STO was highly variable among 11 radiologists and 6 surgeons (kappas ranging from 0.19-0.81). Agreement was substantially better when the STO was applied in a dichotomized way (lower tumour border below STO = rectum, lower tumour above STO = sigmoid), compared to the original 3-way definition by the d'Souza which classified tumours as above, straddling or below the STO. Using the two-way classification >80% consensus could be reached on location in the rectum versus sigmoid colon in 63% of cases (72% for more experienced radiologists).

A study from China (Li 2020, n=635 cancer patients and 200 controls) showed that the distance from the anal verge to the STO was similar between cancer patients and controls, suggesting that it is a reproducible landmark. Compared to other landmarks such as distance in cm from the anal verge and relation to the sacral promontory, use of the STO resulted in more tumours being classified as sigmoid cancer (18% on MRI, 30% on CT).

The sigmoid take off has been adopted as a recommended landmark by SAR DFP recommendations (Lee 2023). There is no evidence yet on the impact of adoption of the STO on long-term patient outcomes.

In summary, the STO appears to be a moderately to well reproducible landmark to discern rectal from sigmoid cancers on imaging and its use is recommended by experts in the field. Based on data from the Netherlands and China, use of the STO may impact tumour classification as rectal versus sigmoid (and consequently also potentially treatment planning, depending on local treatment guidelines) compared to use of alternative/traditional landmarks in ±20-30% of patients. A dichotomized classification offers the most reproducible results.

- D'Souza et al. Definition of the Rectum An International, Expert-based Delphi Consensus. Ann Surg 2019;270:955-959
- Hazen et al. Evaluation of the implementation of the sigmoid take-off landmark in the Netherlands. Colorectal Disease 2022;24:2929-307
- Hazen et al. Impact of the new rectal cancer definition on multimodality treatment and interhospital variability: Results from a nationwide cross-sectional study. Colorectal disease 2024;26:1131-1144
- Hazen et al. An updated evaluation of the implementation of the sigmoid take-of landmark 1 year after the official introduction in the Netherlands. Techniques in Coloproctology 2023;27:1243-1250
- Burghgraef et al. Implications of the new MRI-based rectum definition according to the sigmoid takeoff: multicenter cohort study. BJS Open 2023;7:zrad018
- Bogveradze et al. The sigmoid take-off as a landmark to distinguish rectal from sigmoid tumours on MRI: Reproducibility, pitfalls and potential impact on treatment stratification. Eur J Surg Oncol 2021
- Li et al. Comparison of the sigmoid take-off with other definitions of the rectosigmoid junction: A retrospective comparative cohort analysis. Int J Surg 2020;80:168-174

46. Benefit of classifying the degree of mucin

Draft statement 52

It is recommended to classify a tumour as solid, partly mucinous, or mucinous

(evidence level II)

Summary of relevant literature:
Kim et al. investigated the influence of mucin proportion (relative to total tumour volume; n=27) in rectal cancer on response to neoadjuvant CRT and long-term outcome. Patients with >/=30% mucin at baseline had a worse response than patients with <30% mucin proportion. Patients with low mucin proportion had better prognosis (borderline statistically significant, probably due to sample size).

Oberholzer et al. qualified rectal tumours as mucinous when they had >50% mucinous components (n=21), which had worse response than non-mucinous tumours (which had <50% mucinous components; n=67). Only mucinous tumours showed progression after CRT.

In summary, there is no compelling evidence supporting the reporting of specific percentages of mucin. As a practical approach, we suggest to visually estimate whether tumours are solid, partly mucinous (=solid with some mucinous components), or mucinous (= completely or predominantly mucinous).

- Kim et al. Impact of Mucin Proportion in the Pretreatment MRI on the Outcomes of Rectal Cancer Patients Undergoing Neoadjuvant Chemoradiotherapy. Cancer Res Treat 2019;51:1188-1197. doi: 10.4143/crt.2018.434.
- Oberholzer et al. Rectal cancer: mucinous carcinoma on magnetic resonance imaging indicates poor response to neoadjuvant chemoradiation. Int J Radiat Oncol Biol Phys 2012; 82:842-848. doi: 10.1016/j.ijrobp.2010.08.057

47-48. - How to classify rectal tumour location on MRI (e.g., as distal/mid/high or distal/high) - Use of the anorectal junction versus anal verge? - Are there additional methodological recommendations?

Draft statement 53:

There are no evidence-based definitions or methods detailing how to measure tumour height or classify tumours as distal, mid or high on MRI.

Aiming to enhance uniformity in reporting, ESGAR proposes the following:

- Measure the distance between the lower border of the tumour and the anorectal junction (optionally the distance from the anal verge may be reported as an add on)
- Tumours with a lower border starting <5 cm from the anorectal junction are classified as distal; tumours starting ≥5 cm are together classified as mid-high
- Measure using one or more straight lines following the center of the lumen

(evidence level V)

Summary of relevant literature:
A recent systematic review by Goedegebuure et al. explored practice variations in tumour localization and categorization between various (inter)national clinical and radiological guidelines. Most guidelines use a 3-way classification (high, mid and low); a minority uses a dichotomous classification (distal or high). However, when looking at treatment implications, it is mainly distal location that has a direct impact on treatment stratification.

Landmarks and measurement cut offs to define these subgroups differ between guidelines. The anal verge is the most commonly used landmark (especially in clinical guidelines), but the anorectal junction is also commonly used, especially by radiologists. A commonly used definition for distal rectal cancer is a lower tumour border starting within 5 cm from the anal verge (or 4 -5 cm from the anorectal junction). A minority of guidelines use anatomical landmarks (e.g. above or below peritoneal reflection) rather than distance measurements to establish tumour height.

Methods used in literature to measure tumour height are variable and only a few guidelines provide specific measurement instructions. Both rectilinear and curvilinear measurements have been proposed, but there is no convincing evidence to recommend either type of measurement. Overall, statistical agreement between MRI and endoscopic measurements is good, though different classifications (i.e. distal, mid, or high) may occur in more than half of the cases, depending on the chosen methodology.

In summary, there is substantial variation in literature (incl. guidelines) on the preferred method to define rectal tumour height. Goedegebuure et al. concluded that – at least at an

institutional level – it is important to clearly establish which landmarks and methods are used to ensure consistent MDT discussions and treatment decision-making. They also state that future guideline updates would benefit from updated (and preferably uniform) definitions, accompanied by clear and detailed recommendations on the preferred measurement methodology. As such, as a practical guideline we propose the recommendations outlined in the draft statement.

- Goedegebuure et al. Defining the tumour location in rectal cancer – practice variation and impact on treatment decision making (accepted pending revisions for publication in Eur J Surg Oncol 2025)

---

49. Use of Radiomics and other AI models for (re)staging

Draft statement 54

Radiomics and/or deep learning models should not be used to guide clinical decision making

(evidence level III)

Summary of relevant literature:
As outlined in a recent systematic review (Kim 2024) there are many relatively small studies evaluating some form of radiomics or AI to address a variety of a clinical questions in rectal cancer, related to segmentation, staging, genotyping and response prediction/evaluation. Unfortunately, many of these studies have small samples, lack external validation and are based on retrospective data.

A recent systematic review (Boldrini 2024) summarizes imaging-based prediction models to predict complete response (pCR) to neoadjuvant treatment, focusing on studies that have been externally validated. Most studies show that by combining clinical and radiomics features a better performance is achieved than by using radiomics alone. A systematic review by He et al. confirmed these results and also pointed out that deep learning models may outperform machine learning models.

In summary, literature has shown promising results for Radiomics/AI but results still need to be validated within the context of clinical trials. Results are to date still too immature to base clinical decisions on.

- Boldrini et al. Response prediction for neoadjuvant treatment in locally advanced rectal cancer patients-improvement in decision-making: A systematic review Eur J Surg Oncol 2024;:109463. doi: 10.1016/j.ejso.2024.109463.
- He et al. Machine learning in predicting pathological complete response to neoadjuvant chemoradiotherapy in rectal cancer using MRI: a systematic review and meta-analysis. Br J Radiol 2024;97:1243-1254. doi: 10.1093/bjr/tqae098.
- Kim et al. Performance reporting design in artificial intelligence studies using image-based TNM staging and prognostic parameters in rectal cancer: a systematic review. Annals of Coloproctology 2024;40(1):13-26. DOI: 10.3393/ac.2023.00892.0127
